# Coordination-induced exfoliation to monolayer Bi-anchored MnB_2_ nanosheets for multimodal imaging-guided photothermal therapy of cancer

**DOI:** 10.7150/thno.39715

**Published:** 2020-01-01

**Authors:** Zhaokui Jin, Danyang Chen, Penghe Zhao, Yanyuan Wen, Mingjian Fan, Gaoxin Zhou, Yingshuai Wang, Qianjun He

**Affiliations:** Guangdong Provincial Key Laboratory of Biomedical Measurements and Ultrasound Imaging, National-Regional Key Technology Engineering Laboratory for Medical Ultrasound, School of Biomedical Engineering, Health Science Center, Shenzhen University, No. 1066 Xueyuan Road, Nanshan District, Shenzhen 518060, Guangdong, China.

**Keywords:** two-dimensional nanomaterials, photothermal therapy, MBene, nanotheranostics, manganese boride.

## Abstract

**Background:** Rapid advance in biomedicine has recently vitalized the development of multifunctional two-dimensional (2D) nanomaterials for cancer theranostics. However, it is still challenging to develop new strategy to produce new types of 2D nanomaterials with flexible structure and function for enhanced disease theranostics.

**Method:** We explore the monolayer Bi-anchored manganese boride nanosheets (MBBN) as a new type of MBene (metal boride), and discover their unique near infrared (NIR)-photothermal and photoacoustic effects, X-ray absorption and MRI imaging properties, and develop them as a new nanotheranostic agent for multimodal imaging-guided photothermal therapy of cancer. A microwave-assisted chemical etching route was utilized to exfoliate the manganese boride bulk into the nanosheets-constructed flower-like manganese boride nanoparticle (MBN), and a coordination-induced exfoliation strategy was further developed to separate the MBN into the dispersive monolayer MBBN by the coordination between Bi and B on the surface, and the B-OH group on the surface of MBBN enabled facile surface modification with hyaluronic acid (HA) by the borate esterification reaction in favor of enhanced monodispersion and active tumor targeting.

**Result:** The constructed MBBN displays superior NIR-photothermal conversion efficiency (*η*=59.4%) as well as high photothermal stability, and possesses versatile imaging functionality including photoacoustic, photothermal, CT and *T_1_*-wighted MRI imagings. *In vitro* and *in vivo* evaluations indicate that MBBN had high photothermal ablation and multimodal imaging performances, realizing high efficacy of imaging-guided cancer therapy.

**Conclusion:** We have proposed new MBene concept and exfloliation strategy to impart the integration of structural modification and functional enhancement for cancer theranostics, which would open an avenue to facile fabrication and extended application of multifunctional 2D nanomaterials.

## Introduction

Armed with good understanding of ultrathin structure and its fascinating physiochemical properties, researchers have made innovations in the fields of nano-engineering and materials science to develop two-dimension (2D) nanomaterials for promising applications such as electronic, catalysis, energy harvesting and storage, and biomedicine 1-4. By virtue of ultrahigh specific surface area, powerful plasticity and extraordinary physiochemical properties, 2D nanosheets feature prominently in efficient cancer diagnosis and therapy to overcome the intrinsic limitation of conventional theranostic modalities. Many excellent 2D nanomaterials including graphene 5,6, transition-metal dichalcogenides (TMD) 7, layered metal oxides 8, MXenes 9,10, 2D metal-organic frameworks (MOF) 11-13, boron nitride (BN) 14,15 and black phosphorus (BP) 16 have been developed to construct combination therapy platforms, showing the outstanding performance in cancer therapy. Among that, boron and boron-based nanosheets have also sought immense interest for leveraging the rich properties of low density, ability to capture neutrons, thermoelectricity, partial ionic bonding, and superconductivity 17,18. However, the research on 2D boron-based nanosheets has been primarily limited to hexagonal boron nitride and elemental boron. In addition, MXenes as a kind of emerging nanomaterials have exhibited a special sandwich layered structure as well as many unique physicochemical properties which derives their broad applications in the field of energy and biomedicine 19-21, but the current definition and knowledge of MXenes are only limited to metal carbide and nitride. Recognizing that metal boride (MB) has also a kind of the similar sandwich layer molecular structure 22, we here classify MB into MBene 23,24, and hypothesize that MB could be exfoliated into 2D nanosheets as magnesium boride with the similar molecular structure had been exfoliated into nanosheets by us recently, and we also envision that MBN could demonstrate some special optical and magnetic properties which are different from MB bulk and could be utilized for biomedical applications 25-27.

**Scheme 1:**
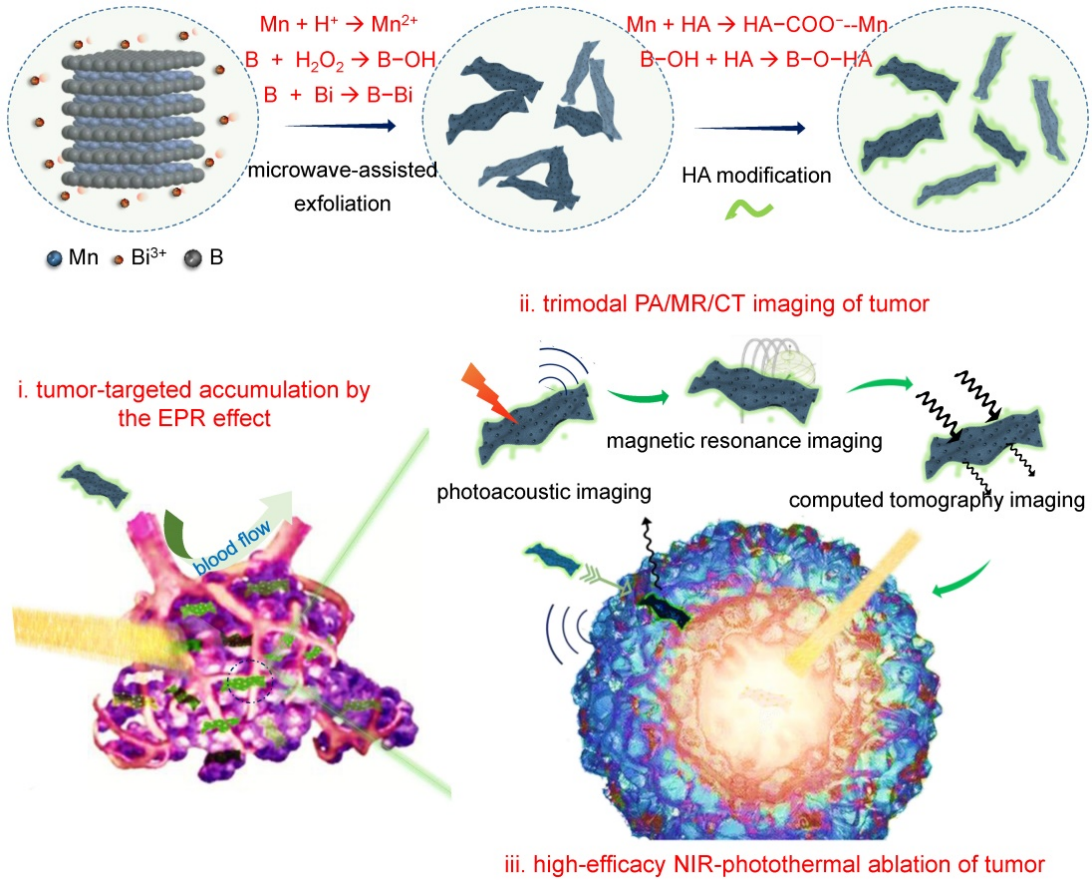
Schematic illustration of the coordination-induced exfoliation for MBBN and the tumor-targeted delivery of MBBN for multimodal imaging-guided NIR-photothermal therapy.

Herein, we developed a microwave-assisted chemical etching method to effectively exfoliate the manganese boride podwer (MBP) into the nanosheets-constructed flower-like manganese boride nanoparticle (MBN), and further developed a coordination-induced exfoliation technique to obtain the Bi-anchored single manganese boride nanosheet (MBBN) by intercalating/reducing bismuth ion and then separating the MBN nanoflower into the dispersive nanosheets (Scheme 1). The monolayer MBBN possessed excellent NIR-photothermal conversion performance (*η*=59.4%) with superior photothermal stability, multiple PA/MR/CT imaging functions, and active and passive tumor-targeting features owing to small nanoscale and facile surface modification with hyaluronic acid (HA), resulting in high outcome of cancer photothermal therapy (PTT) under guidance of PA/MR/CT imagings.

## Results and Discussion

**Figure 1:**
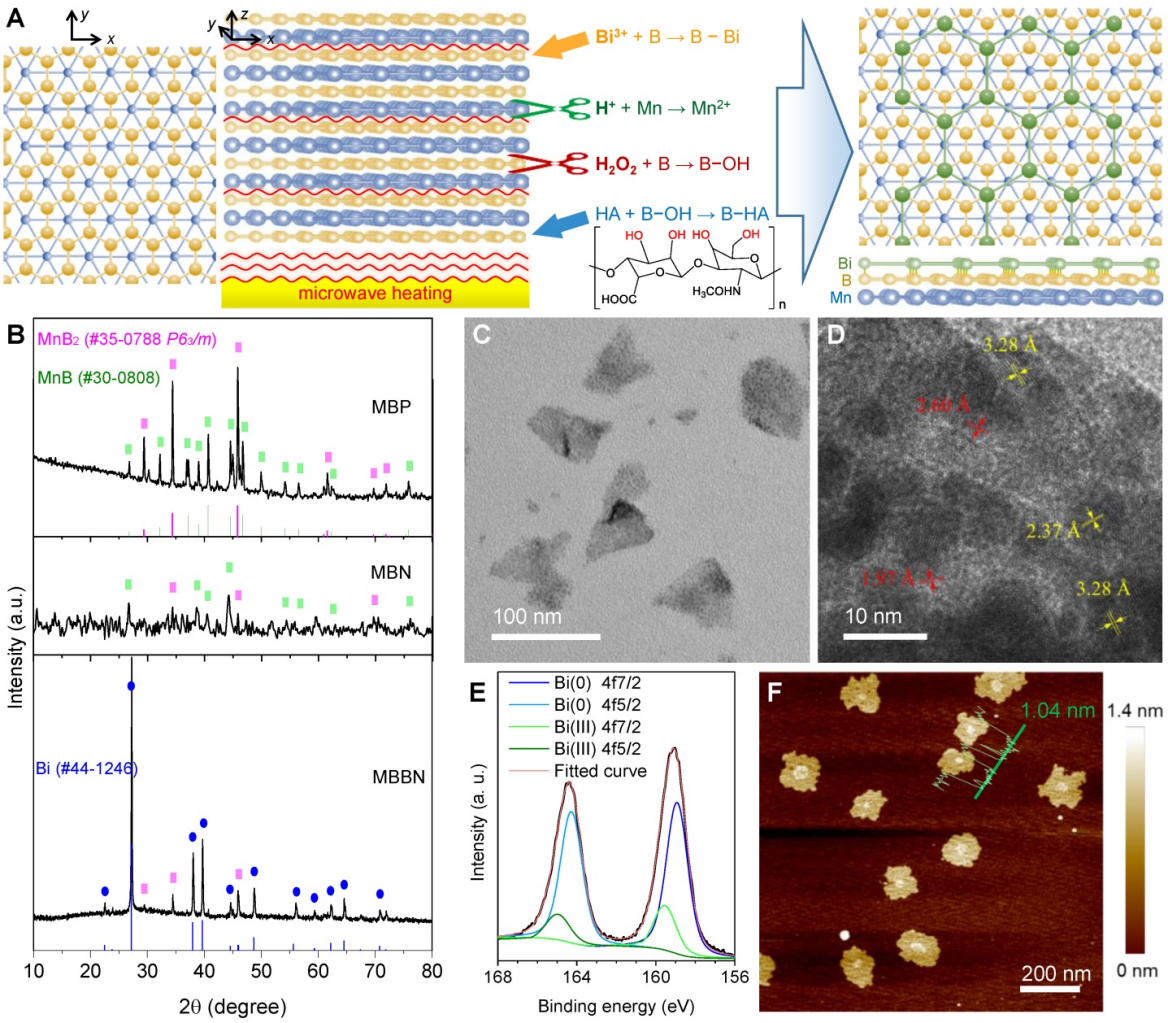
Morphological and microstructural characterizations. (a) Schematic illustration of coordination-induced microwave-assisted chemical exfoliation of MBBN. (b) XRD patterns of MBP, MBN and MBBN. (c,d) Typical TEM image (c) and corresponding HR-TEM image (d) of MBBN. (e) High-resolution XPS spectra of Bi in MBBN. (f) Typical AFM image of MBBN, where the insert green curve represents the height distribution with average value of 1.04 nm.

Generally, MXenes were synthesized by the chemical etching method where chemical etching agents were used to etch off one of layers within ABC-type trielemental layered compounds 28,29. Typically, the Al layer was facilely removed from Ti_3_AlC_2_ by NH_4_HF_2_ to obtain the Ti_3_C_2_ nanosheet 30. However, the same method is hard to exfoliate metal boride bulk into nanosheets because metal boride has only two elemental layers and the interlayer connection relies on intense ionic bonding which leads to superhard property. Therefore, we used acid and H_2_O_2_ as two etching agents to exfoliate metal and boron layers under the assistance of microwave heating, respectively, and also to break the interlayer metal-boron binding (Figure 1a). By such a microwave-assisted chemical etching method, the manganese boride podwer (MBP) was successfully exfoliated into a kind of flower-like structure consisted of nanosheets (Figure S1a). The commercial MBP included two crystal phases, MnB_2_ (JCPDS card no. 35-0788) and MnB (JCPDS card no. 30-0808), which caused that the obtained manganese boride nanosheet (MBN) also contented these two phases (Figure 1b). When Bi^3+^ was added in the etching reaction system for synthesis of MBBN, the MnB phase disappeared while the Bi(0) phase was yielded (Figure 1b-e), indicating that there might occur a redox reaction: MnB + Bi^3+^ → MnB_2_ + Bi + Mn^2+^. Moreover, the emergence of a part of Bi(III) suggested the formation of coordinated B-Bi bonding on the surface of MnB_2_ nanosheets 31,32, leading to the stable anchoring of Bi nanocrystals on the MnB_2_ nanosheet as demonstrated in Figure 1c-e and Figure S2. It was also worth noting that the coordination, reduction and anchoring of Bi on the surface of MBN caused the destruction of flower-like nanostructure into single nanosheets (Figure S1, Figure S3, Figure 1c). From the AFM pattern in Figure 1f, the average thickness and diameter of the MBBN were calculated to be about 1.04±0.34 nm and 150 nm, respectively, implying the MnB_2_ nanosheet was single B-Mn-B layer (0.7 nm thick in theory) which could also be reflected by the emerging Vis-NIR absorption after Bi-assisted exfoliation (Figure S4). From HR-TEM (Figure 1d) and AFM data (Figure 1f), it could be further found that Bi nanocrystals were a kind of ultrathin monatomic layer nanosheet with thickness and diameter of about 0.34 nm (equal to the diameter of a Bi atom) and 10 nm, respectively. It was quite interested that the B layer on the surface of MnB_2_ could well be matched with the Bi layer (Figure 1a) because of the same hexagonal honeycomb-like structure and the quite approximate parameter of crystal cell (*a*=0.2778 nm for MnB_2_ and *a*=0.4545 nm for Bi), which could be one of main causes for the anchoring of monatomic Bi layer on the surface of single MnB_2_ layer besides the coordination attraction between Bi and B.

By ICP measurement (Table S1), it was found that the coordination amount of Bi could reach Bi:Mn=0.049 mol/mol for MBBN. When we attempted to increase the Bi content, a part of Bi was oxidized into Bi_2_O_3_ from XRD (JCPDF no.27-0052, Figure S5) and XPS (160.4 and 165.8 eV) patterns, which increased with the increase of Bi source amount (Figure S6). AFM patterns in Figure S7 indicated that the formation of excessive Bi_2_O_3_ increased the thickness of MnB_2_ nanosheet and also destructed its morphology from TEM images (Figure S1), violating the purpose of this work. The further ultrasound-assisted modification with hyaluronic acid (HA) could improve biocompatibility and dispersibility of MBBN at desirable nanosize. In addition, ATR-IR results suggested that the B-OH group was clearly formed on the surface of MBN and MBBN, as reflected by the emerging peaks at 862 cm^-1^ (purple zone, Figure S8), which enabled the modification with polyalcohols such as tumor targeting molecule HA to form borate (gray zone, Figure S8) and coordination (Scheme 1) in favor of particle dispersion and long-term stability as suggested by DLS data (Figure S9).

**Figure 2:**
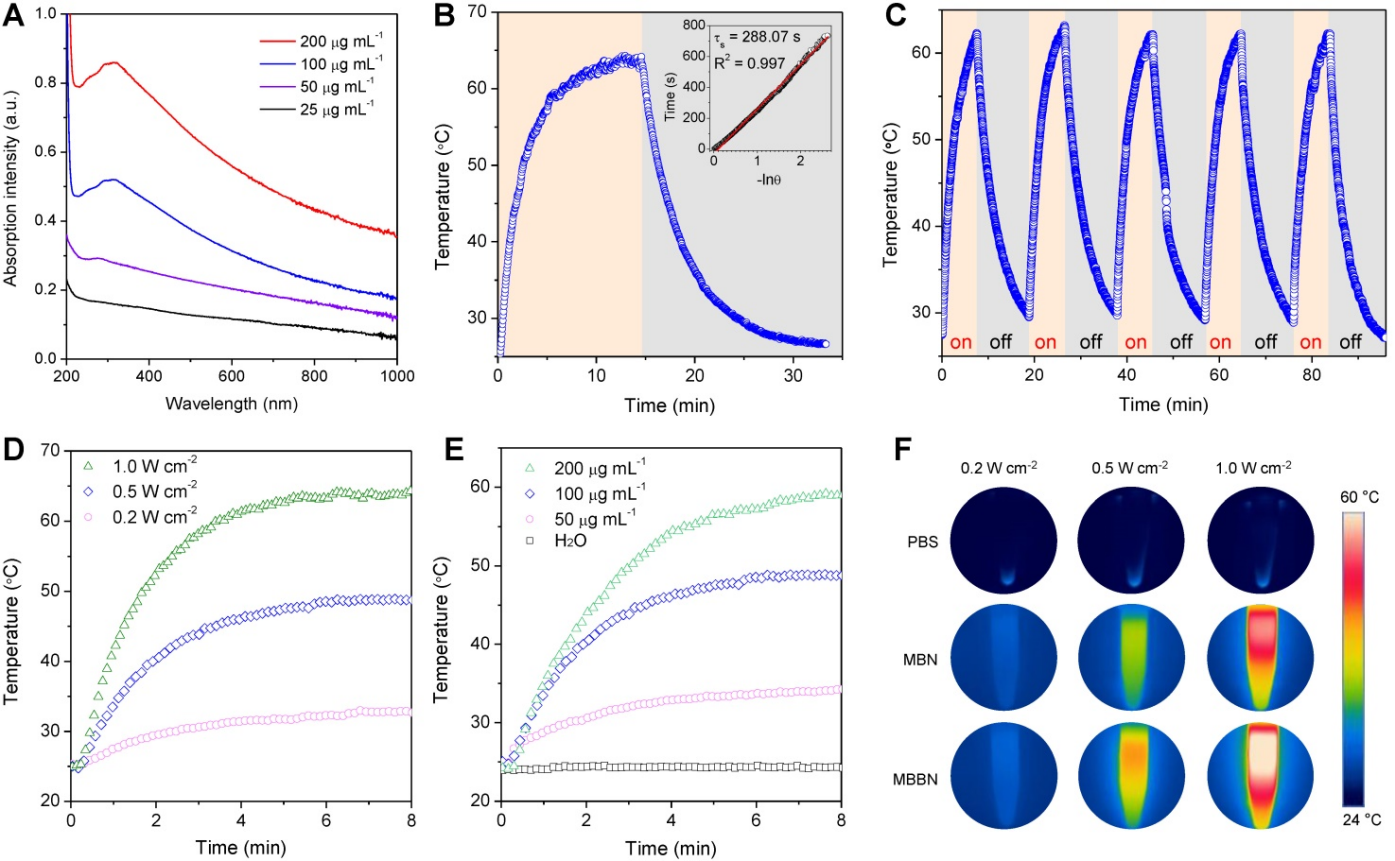
Photothermal conversion performance and stability of MBBN. (a) UV-VIS-NIR absorbance spectra of MBBN at varied concentrations (25, 50, 100, 200 μg mL^-1^). (b) Calculation of the photothermal-conversion efficiency of MBBN (100 μg mL^-1^) under the irradiation with 808 nm laser (1.0 W cm^-2^). The insert image is the linear relationship between time and -lnθ calculated from cooling period after the laser was turned off. (c) Photothermal conversion stability of MBBN for five laser on/off cycles (1.0 W cm^-2^). (d) Photothermal heating curves of MBBN (100 μg mL^-1^) under the irradiation with 808 nm laser at varied power densities (0.2, 0.5, 1.0 W cm^-2^). (e) Photothermal heating curves of deionized water and MBBN at different concentrations (50, 100, 200 μg mL^-1^) under the same irradiation with 808 nm laser at the power density of 0.5 W cm^-2^. (f) Thermal images of MBN and MBBN (100 μg mL^-1^) after 5 min 808 nm laser irradiation (0.2, 0.5, 1.0 W cm^-2^).

Inspired by the unique single layer structure and UV-Vis-NIR adsorption property (Figure S4), we hypothesized that the MBBN could be developed as a NIR-photothermal agent for photothermal therapy and photoacoustic imaging. As a photothermal agent, its extinction coefficient and photothermal conversion efficiency (*η*), which could reflect the abilities of absorbing light and converting light energy into thermal energy, respectively, are two decisive parameters that determine its hyperthermia performance. From Figure 2a, the MBBN showed a board absorption band from UV to NIR with varying concentration and its absorbance depended on its concentration, which is similar to many typical 2D nanomaterials such as graphene and MXenes 33-35. Furthermore, the NIR-photothermal conversion efficiency (η) of MBBN under the irradiation of 808 nm laser at the power density of 1.0 W/cm^2^ was measured by the Roper method 36,37 (see details in the Supporting Information) to be 59.4% (Figure 2b) with a slight influence by the power density (Figure S11), which was much higher than that of MBN (41.6%, Figure S12) owing to the additional contribution of Bi, and also is remarkably higher than that of other reported MXenes (30.6% for Ti_3_C_2_, 44.7% for Ta_4_C_3_, 36.4% for Nb_2_C) 34,38,39. Moreover, both the NIR-photothermal outcome of MBN and MBBN demonstrated high stability (Figure 2b and Figure S13), ensuring on-demand and sustainable photothermal therapy and photoacoustic imaging. Such an outstanding NIR-photothermal feature reminded us that the MBBN could be indeed developed as NIR-photothermal agent.

The photothermal performance was further evaluated *in vitro* by irradiating the aqueous solution of MBBN with different concentrations (0-200 μg mL^-1^) by an 808-nm laser at varied power densities (0.5-1 W cm^-2^). In contrast to the negligible temperature increase of pure water, relatively low concentration of MBBN (100 μg mL^-1^) presented a high temperature increase (Δ*T*) of 22.7 °C after 0.5 W cm^-2^ NIR laser irradiation for 5 min (Figure 2c), suggesting distinct NIR-photothermal effect of MBBN. From Figure 2d and Figure S10, the photothermal effect of MBBN and MBN was dependent on both the concentration of particle and the power density of light, so that the temperature could increase by 38 °C after 1 W cm^-2^ NIR irradiation of 200 μg mL^-1^ MBBN for 5 min (Figure 2de). Moreover, the NIR-photothermal effect of MBBN was more remarkable than that of the MBN (Figure 2f), owing to higher photothermal conversion efficiency.

**Figure 3:**
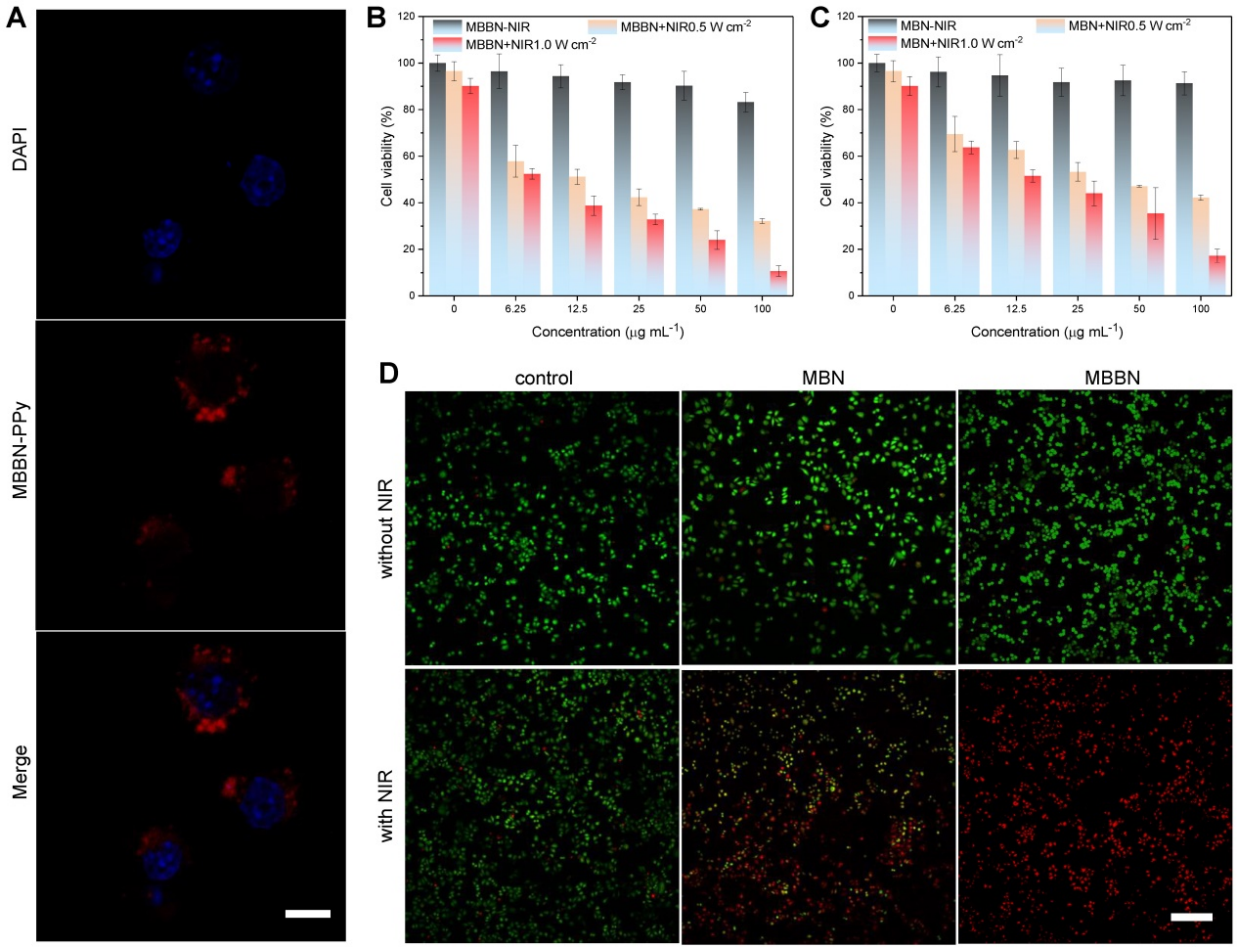
*In vitro* assessments of MBBN-induced photothermal ablation of cancer cells. (a) CLSM images of 4T1 cells after co-incubation with MBBN-PPy for 4 h (MBBN-PPy, 50 μg mL^-1^, red fluorescence; DAPI, blue fluorescence). Scar bar=10 μm. (b,c) Relative viabilities of 4T1 cells after treatment with varied concentrations of MBBN (b) or MBN (c) at different laser power densities (0, 0.5, 1.0 W cm^-2^). (d) The corresponding CSLM images (scale bar, 100 μm for all the panels) of 4T1 cells stained with calcein AM (live cells, green fluorescence) and PI (dead cells, red fluorescence) after different treatments.

The *in vitro* cytotoxicity of MBN and MBBN to cancer cells was measured using a standard CCK-8 assay and their NIR-photothermal therapy effects were evaluated. From CSLM images in Figure 3a, it could be found that the MBBN was easily took up by 4T1 cells after 4 h incubation, owing to small particle size and favorable nanosheet morphology 40,41 as well as active recognization effect of HA which is the ligand of CD44 frequently overexpressed on many kinds of cancer cells such as 4T1 and BGC-823 cells 42,43. From Figure 3b-c, Figure S14 and Figure S15, there was no significant cytotoxicity of MBN and MBBN to various normal and cancer cells (HEK-293T, 4T1 and BGC-823 cells) in a wide concentration range of 6.25-100 μg mL^-1^ in the absence of NIR irradiation, indicating low cytotoxicity and high potential in the bioapplication. However, 5 min NIR irradiation of MBN and MBBN could induce the obvious apoptosis of 4T1 cells as indicated by live/dead cell staining results in Figure 3d, and the MBBN with NIR irradiation exhibited remarkably higher cytotoxicity than the MBN in accordance with CCK-8 results (Figure 3b-c), due to stronger NIR-photothermal effect of MBBN (Figure 2f). In addition, both higher particle concentration and higher power density of NIR light could cause higher cytotoxicity to both 4T1 cells and BGC-823 cells because of the concentration-/power-dependant effect of MBBN (Figure 2d-e). More than 73% of 4T1 cells and up to 83% of BGC-823 cells were effectively killed by 100 μg mL^-1^ MBN under 1 W cm^-2^ NIR irradiation (Figure S16). By contrast, MBBN could kill 89% of 4T1 cells and 95% of BGC-823 cells at the same conditions. These results demonstrated that MBBN had high efficiency of *in vitro* photothermal cancer therapy.

**Figure 4:**
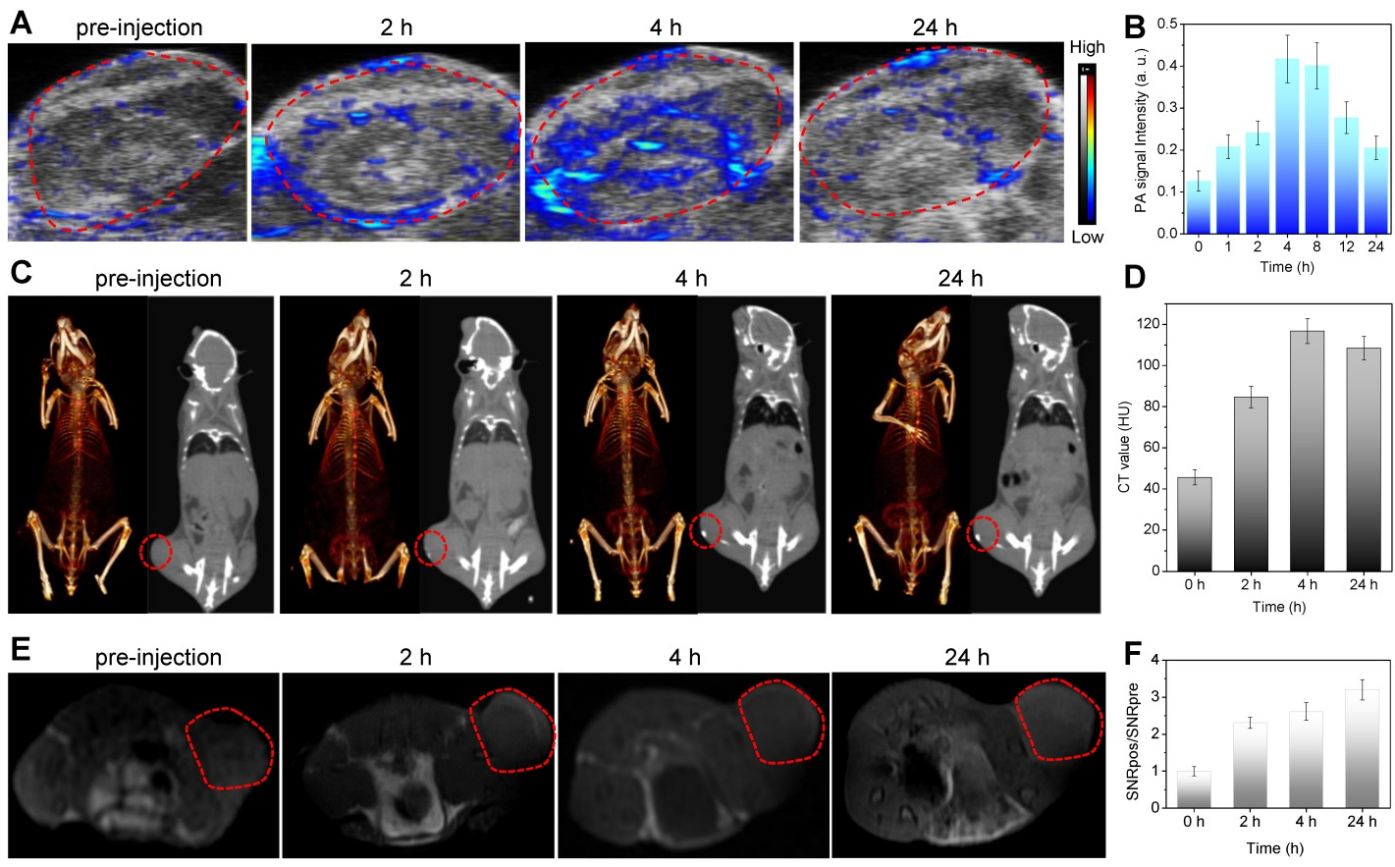
*In vivo* multimodal imaging of MBBN. (a) *In vivo* PA images acquired from 4T1 tumor-bearing Balb/c mice after intravenous injection of MBBN (2 mg mL^-1^, 100 μL) at different time points (0, 2, 4, and 24 h) post-injection. (b) Corresponding PA signal analysis of the tumor in figure a. (c) *In vivo* CT contrast (right) and 3D reconstruction CT (left) images of tumor-bearing mice before and after intravenous injection of MBBN (6 mg mL^-1^, 100 μL). (d) Corresponding CT contrasts analysis of the tumor in figure c. (e) *In vivo* MR images of the tumor-bearing mice at different time points after intravenous injection of MBBN (2 mg mL^-1^, 200 μL). (f) Corresponding MR signal analysis of the tumor in figure e. All the red circles mark the tumors.

Besides the efficient suppression in fighting cancer, tackling some of the critical issues confronting diagnostic imaging by nanomaterials is highly significance for evaluating the nanotheranostics. The complementary abilities of different imaging modalities could be harnessed to advocate effectively for the resolution and sensitivity. For example, the combination of CT and MRI are cited to be desirable for image-guided therapy where CT is used to perform electron-dense elements mapping and MRI provides soft tissue contrast to identify target tissues 44,45. Therefore, the combination of CT, MRI and PAI (higher sensitivity and temporal resolution, but lower penetration depth) 45,46 could integrate their advantages and compensate the shortcomings of individual imaging mode to obtain more biomedical information. Furthermore, the corresponding imaging contrast agents could be used to enhance imaging performances, and the integration of multiple imaging and therapy functions into a single nanoparticle could realize nanotheranostics in favor of non-invasive dynamical monitoring of nanomedicine accumulation and accurate guidance of therapy. Bi has high X-ray attenuation ability due to its high atomic number (Z = 83), and thus could be used for CT imaging 47-49. Therefore, we conjugated Bi onto MBN to construct MBBN for multimodal CT/MR/PA imaging-guided photothermal therapy of cancer. *In vitro* measurement suggested that the MBBN possessed excellent CT/MR/PA imaging performances with the X-ray absorption coefficient of 28.6 HU mL mg^-1^ (Figure S19), the proton relaxivity *r_1_* of 0.794 s^-1^ mL mg^-1^ (Figure S20), and the photoacoustic coefficient of 0.908 mL mg^-1^ (Figure S18). To explore the feasibility of *in vivo* using MBBN as a multimodal imaging contrast agent, we collected the PA/CT/MR signals of 4T1 tumor-bearing mice before and after intravenous injection of MBBN (100 μL, 6 mg mL^-1^). From Figure 4, it could be found that the tumor of mice treated with MBBN could be clearly imaged by PA/CT/MR modes, verifying the tumor-targeting and *in vivo* PA/CT/MR imaging abilities of MBBN. By real-time monitoring of intratumoral accumulation of MBBN by PA/CT/MR imagings, the process of gradual intratumoral accumulation of MBBN was discovered and the intratumoral accumulation of MBBN could reach the maximum at about 4 h after intravenous injection. These biomedical imaging informations would provide great help for guidance of NIR-triggered therapy of cancer.

**Figure 5:**
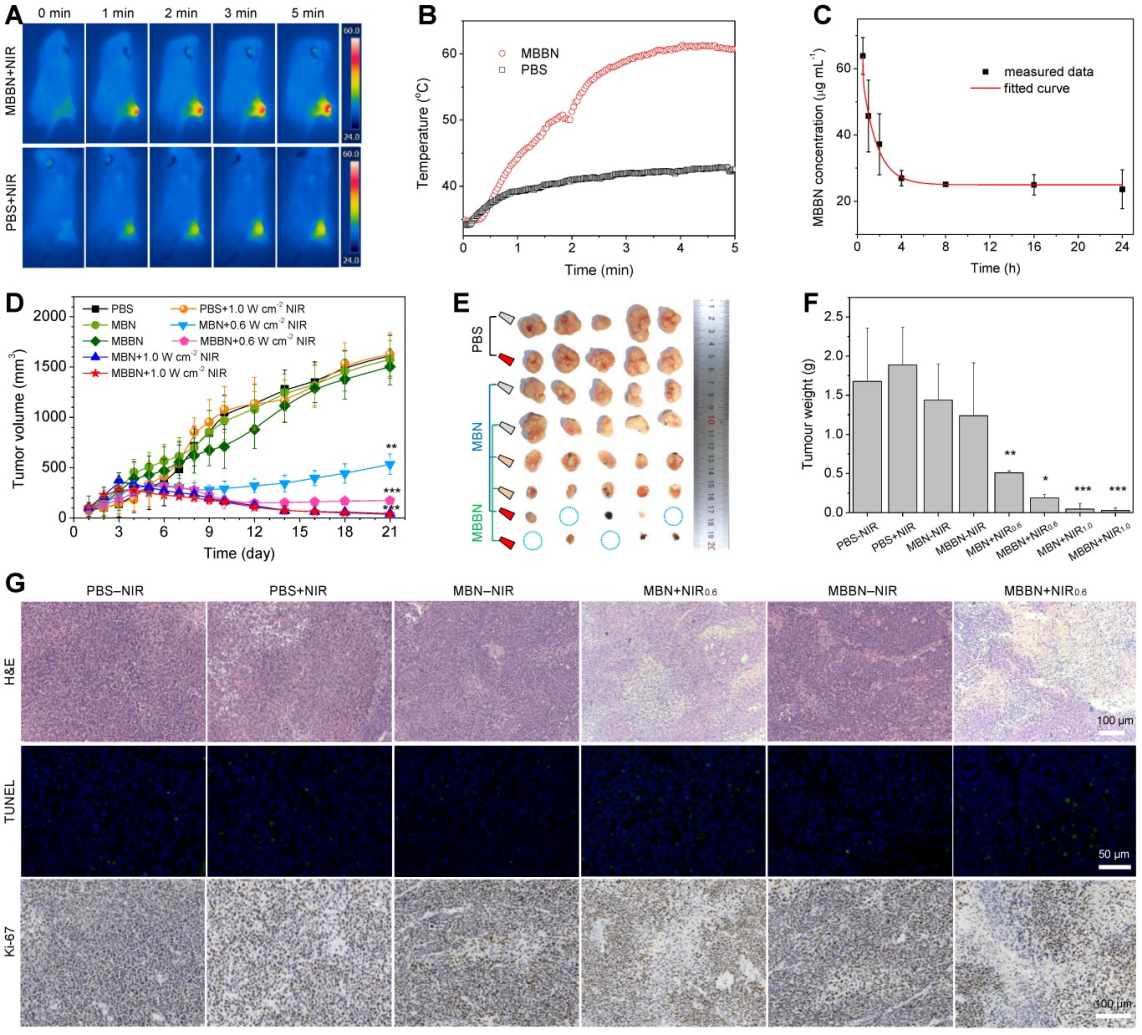
MBBN-mediated photothermal cancer therapy *in vivo*. (a) Infrared thermographic images of 4T1-tumor-bearing mice in groups of PBS (100 μL) and MBBN (20 mg kg^-1^) at different time points during laser irradiation. (b) The corresponding time-dependent temperature changes at the tumor sites of 4T1-tumor-bearing mice during laser irradiation. (c) Time-dependent tumor growth curves of BGC-823 tumor-bearing nude mice (*n*=5, mean ± s.d.) in groups of PBS-NIR control, PBS+NIR, MBN-NIR, MBN+NIR_0.6_, MBBN-NIR, MBBN+NIR_0.6_, MBN+NIR_1.0_, MBBN+NIR_1.0_ during laser irradiation. (d) Digital photos of tumors harvested from mice in different groups after 21 days of treatment. (e) The corresponding tumor weight of mice in different groups after 21 days of treatment. *P* values were calculated by two-tailed Student's *t*-test (***P<0.005). (f) Histological analysis including H&E staining for pathological changes in tumor tissues to reveal the effectiveness of *in vivo* photothermal therapy, the TUNEL staining for cell apoptosis in tumor sections, and Antigen Ki-67 immunofluorescence staining for cellular proliferation in tumor sections.

Based on above imaging information, the blood circulation and biodistribution of MBBN were further investigated before the PTT treatment *in vivo*. From Figure 5c, the circulation of MBBN in bloodstream was measured to abide by a compartment model, and the blood circulation half-time of MBBN was calculated to be about 1.1 h. The biodistribution of MBBN in normal organs and tumor was observed by real-time fluorescence imaging. As shown in Figure S21, both *in vivo* and *ex vivo* imaging results showed that MBBN possessed excellent tumor-targeted accumulation property. Long blood circulation and efficient intratumoral accumulation of MBBN were in favor of high-efficacy photothermal therapy. Encouraged by above results, after intravenous injection of MBBN (20 mg kg^-1^ PBS solution) for 4 h, the tumor-bearing mice were anesthetized and their tumor sites were exposed to 808 nm laser at a power density of 1.0 W cm^-2^ to evaluate the effect of NIR-photothermal therapy of cancer. From Figure 5a-b, the temperature in the tumor site of mice injected with MBBN rapidly increased from 33°C to 60°C after 5 min NIR irradiation, which was much more remarkable than blank control and MBN (injection of PBS or MBN plus NIR, Figure S22), owing to higher intratumoral accumulation and higher NIR-photothermal effect of MBBN. Such a visible NIR-photothermal effect of MBBN *in vivo* enabled cancer therapy. We then performed *in vivo* photothermal therapy on BGC-823 tumor-bearing nude mice. When the subcutaneously inoculated tumors reached around 100 mm^3^, the nude mice were randomly divided into eight groups (*n*=5): PBS-NIR (50 μL), PBS+NIR (50 μL), MBN-NIR (2 mg mL^-1^, 50 μL), MBN+NIR_0.6_ (2 mg mL^-1^, 50 μL), MBN+NIR_1.0_ (2 mg mL^-1^, 50 μL), MBBN-NIR (2 mg mL^-1^, 50 μL), MBBN+NIR_0.6_ (2 mg mL^-1^, 50 μL), MBBN+NIR_1.0_ (2 mg mL^-1^, 50 μL). The 5 min irradiation with 808 nm laser at a power density of 0.6 or 1.0 W cm^-2^ was carried out twice at 1 h and 25 h post-injection. After treatment for 21 days, all the treated mice survived, possibly owing to low lethality of used tumor model within 21 days and high bio-safety of used nanomedicines. From the collected everyday record by a digital caliper (Figure 5d and Figure S23), neither MBN-NIR nor MBBN-NIR groups induced tumor suppression compared to the PBS-NIR group, in which the tumor volumes reached up to 1000 mm^3^ after 21 days treatment. By comparison, four photothermal groups (MBN+NIR_0.6_, MBN+NIR_1.0_, MBBN+NIR_0.6_, MBBN+NIR_1.0_) showed effective inhibition of tumor growth (Figure 5d-f) in a power-dependent manner, and even completely eradicated the tumors of some mice as indicated by blue circles in Figure 5e and Figure S27 under the power density of 1.0 W cm^-2^. At the power density of 0.6 W cm^-2^, MBBN exhibited remarkably higher tumor suppression efficacy than MBN, due to its higher photothermal conversion efficiency. All the mice demonstrated negligible weight fluctuation (Figure S23), indicating insignificant adverse effects of these administrations on the health of mice.

The effective photothermal treatment was further confirmed by post-mortem histological analysis. From the hematoxylin and eosin (H&E) staining results (Figure 5g, Figure S24), two photothermal groups showed significantly reduced cell density as well as damaged architectures in tumors, while the control groups (PBS-NIR, PBS+NIR, MBN-NIR, MBBN-NIR) displayed densely packed neoplastic cells. In addition, TUNEL staining results indicated the obvious increase in green fluorescence in two photothermal groups (MBN+NIR, MBBN+NIR), suggesting severe necrosis of tumor cells by the photothermal ablation of MBN and MBBN. Antigen Ki-67 staining was also employed to evaluate the proliferative activities of tumor cells. The results showed that the two photothermal groups presented strong suppression effect on the tumor cell proliferation compared to control groups.

To further evaluate *in vivo* translation potential of MBBN, a detailed investigation of *in vivo* toxicology was conducted. Fifteen Balb/c mice were randomly divided into three groups (*n*=5) for intravenous injection of PBS, MBN, MBBN (2 mg mL^-1^, 100 μL) and then collected the blood from mice orbit after one week. The general haematology parameters (red blood cells RBC, white blood cells WBC, haemoglobin HGB, haematocrit HCT, mean corpuscular haemoglobin MCH, mean corpuscular haemoglobin concentration MCHC, platelets PLT and mean corpuscular volume MCV) and standard blood biochemical indexes (alanine transaminase ALT, aspartate transaminase AST, total protein TP, globulin GLB, total bilirubin TBIL, blood urea nitrogen BUN, creatinine CREA and albumin ALB) were measured. From the Figure S26, there was no statistically significant difference observed from both the haematology parameters and blood biochemical indexes of MBN and MBBN in comparison to the control group. These results demonstrated that the treatment with MBN and MBBN did not generate negative impact on physiological toxicity and blood chemistry. Besides, the corresponding histological changes of major organs, including the heart, liver, spleen, lung and kidney, were collected after *in vivo* therapy and sliced for H&E staining (Figure S25). It showed no obvious acute, chronic pathological toxicity and adverse effects after 21 day treatment for all groups, indicating no significant histological abnormality in the treatment group.

## Conclusion

In conclusion, we have successfully demonstrated that manganese boride can be contrivablely exfoliated to metal boride nanosheet as a new type of MBene, and be further harnessed to multifunctional nanotheranostics by coordinated-induced construction of monolayer Bi-anchored manganese boride nanosheets (MBBN). By virtue of microwave-assisted chemical etching, a coordination-induced exfoliation strategy was developed to fabricate ultrathin MBBN at average size of 150 nm and 1.04 nm in depth with the coordination between Bi and B on the surface, which enabled facile surface modification with hyaluronic acid (HA) by the borate esterification reaction in favor of biocompatibility. The constructed MBBN exhibited strong NIR absorption and efficient NIR-induced photothermal performance with a high photothermal conversion efficiency of 59.4%, as well as excellent photothermal stability and high biocompatibility supported by *in vitro* and *in vivo* toxicity study. Utilizing the excellent photoacoustic effects, X-ray attenuation and *T_1_*-weighted ability of MBBN, the integration of largely enhanced *in vivo* PA/CT/MR imaging of tumors have been achieved. Significantly, outstanding positive outcomes were achieved by the utilization of MBBN as phototheranostics for *in vitro* and *in vivo* photothermal ablation in tumor xenografts. Our study proposes metal boride nanosheets as a new type of MBene, which can be integrated with a wide range of functionalities, biodegradability and biocompatibility for cancer nanotheranostics.

## Materials and Methods

### Preparation of MnB nanosheets (MBN)

150 mg of raw MnB powders and 450 mg PVP were dispersed in 40 mL of EG. Then, 4 mL of H_2_O_2_ (30%) and 400 μL of CH_3_COOH were added in sequence to the solution. The mixture was transferred to an autoclave and heated at 160 °C for 2h using a microwave synthesizer ( 400 W, UWAVE-2000). After cooling to room temperature, the product was centrifuged at 14000 rpm, and washed twice with EtOH and DI water, and redispersed in 20 mL of DI water. For the further surface modification of HA, HA was added to the obtained solution with a feed ration of 1:1 (m/m), and sonicated for 1 h (150 W, 4.4 s on/2.2 s off) by a sonictip. The resulting black dispersion was centrifuged at 1000 rpm for 10 min to get rid of bulk MnB, and the supernatant containing MBN was carefully collected at the centrifugation of 14000 rpm.

### Preparation of Bi-anchored MnB nanosheets (MBBN)

The anchoring of Bi was using a similar method as MBN except for the addition of Bi(NO_3_)_3_·5H_2_O as a source of Bi. 150 mg (~1.95 mmol) of MnB powders, 94.6 mg Bi(NO_3_)_3_·5H_2_O (~0.195 mmol) and 450 mg PVP were dispersed in 40 mL of EG. Then, 4 mL of H_2_O_2_ (30%) and 400 μL of CH_3_COOH were added in sequence to the solution. The mixture was transferred to an autoclave and heated at 160 °C for 2h using a microwave synthesizer ( 400 W, UWAVE-2000). After cooling to room temperature, the product was centrifuged at 14000 rpm, and washed twice with EtOH and DI water, and redispersed in 20 mL of DI water. The modification of HA was same to MBN. To investigate the effect of Bi cations on the exfoliation, the amount of Bi(NO_3_)_3_·5H_2_O was varied from 0.1 to 0.4 to obtain several products as MBBN_0.1_, MBBN_0.2_, MBBN_0.4_.

### Photothermal effects of MBN and MBBN

The photothermal heating curves were obtained by monitoring the temperature change of sample solutions under the irradiation of 808 nm laser at different power densities (0.2-1.0 W/cm^2^). The laser was provided by a fiber-coupled continuous semiconductor diode laser (KS-810F-8000, Kai Site Electronic Technology Co., Ltd.) and the temperature was recorded by a fixed-mounted thermal imaging camera (FLIR A300-series).

### Cytotoxicity measurement of the prepared MBN and MBBN

The mouse 4T1 breast cancer cells, human BGC-823 gastric carcinoma cells, and HEK-293T emborynic kidney cells were purchased from China Type Culture Collection (CTCC) obtained from the American Type Culture Collection (ATCC). First, the cancer cell lines and normal cell lines were used to study the dark toxicity of MBN and MBBN. The cells (1×10^4^ cells/well) were planted in the 96-well plate. After incubation for 12 h, the culture medium was replaced with fresh ones containing MBN or MBBN at final concentrations of 6.25-100 μg/mL. After another 24 h incubation, 10 μL CCK-8 solution was added into each well. After incubation for another 0.5 h, the plate was read using the Bio-Tek multi-mode microplate reader (absorption wavelength: 450 nm). To study the *in vitro* therapy effect, the cellular viability was tested by the CCK-8 method *via* different types of treatment as follows. (1) control group: 4 h incubation with MBN (or MBBN) then for another 24 h or 48 h incubation; (2) only PTT group: 4 h incubation with MBN or MBBN irradiated with the 808 nm NIR laser at 1.0 W/cm^2^ or 0.5 W/cm^2^ for 10 min every one well; After incubation for 24 h, the cytotoxicity was performed by the CCK-8 assay. The cytotoxicity was expressed as the percentage of cell viability as compared with the blank control. Each data point was represented as a mean ± standard deviation of five independent experiments (*n* = 5).

### Confocal Fluorescence Imaging

4T1 cells were seeded into a CLSM-special cell-culture dish, which were cultured at 37 °C under 5% CO_2_ atmosphere. Then, 100 μL of MBN or MBBN DMEM solution was added into the dish. After co-incubation for 4 h, the cell solution was irradiated by an 808 nm laser for 5 min at a power density of 1.0 W cm^-2^. After the cells were washed with PBS three times, confocal fluorescence images of calcine AM (green) and PI (red) co-stained cells further confirmed the death of 4T1 cells induced by MBN or MBBN after laser irradiation. To investigate uptake of MBBN by cells, a typical fluorescent dye, 5,10,15,20-Tetra(4-pyridyl)porphyrin (PPy) were firstly labelled on MBBN via the Mn-PPy coordination to obtain MBBN-PPy (as shown in Figure S17). Then, 4T1 cells were seeded into a CLSM-special cell-culture dish, and co-incubated with MBBN (50 μg mL^-1^) for 4 h. After cell fixation and 3-4 times wash with PBS, the cells were stain with DAPI (50 μg mL^-1^) for 15 min, and observed with CSLM.

### *In vivo* photothermal imaging (PTI)

The 4T1 tumor-bearing mice model was established by injecting 1×10^7^ 4T1 cells into the hind limb of each Balb/c mouse (~20 g, purchased from Guangdong Medical Laboratory Animal Center). After the mean volume of the tumors reached about 100 mm^3^, two experienced researchers randomly divided the mice into three groups (*n* = 3 per group). The tumor-bearing mice were injected with 100 μL of PBS (Group 1), or 20 mg kg^-1^ MBN (Group 2), or 20 mg kg^-1^ MBBN (Group 3) through the vein of tail. After 4 h injection, the mice were anesthetized by 4% chloral hydrate (120 μL) and then irradiated with the 808 nm laser at 1 W cm^-2^ for 5 min. During the course of irradiation, we utilized the infrared thermal imaging cameras (FLIR A300-series) to monitor the temperature change of the tumor sites. Group 1 was taken as the control. The Administrative Committee on Animal Research in Shenzhen University approved the protocols for all animal experiments.

### *In vivo* photoacoustic imaging (PAI)

All mouse imaging experiments were performed using a real-time multispectral optoacoustic tomographic imaging system (Vevo 2100 LAZR system, Visual Sonic Inc. New York, NY) equipped with a 40 MHz, 256-element linear array transducer on tumors. For *in vivo* PA imaging study, subcutaneous 4T1 tumor bearing Balb/c mice were anesthetized by 1% isoflurane delivered *via* a nose cone, and then the MBBN (100 μL, 2 mg mL^-1^) were injected *via* the tail vein. *In vivo* PA images were acquired before injection and at different time points post injection (1 h, 2 h, 4 h, 8 h, 12 h and 24 h) using the multispectral optoacoustic tomography system at a wavelength of 780 nm. A region of interest (ROI) volume consisting of transverse slices with a step size of 0.4 mm, spanning through the tumor region, was selected by manual inspection of live MSOT images. The average PA signal of the tumor area was extracted using the multispectral optoacoustic tomography software.

### *In vivo* CT imaging

Two Balb/c 4T1-bearing tumors mice were anesthetized with 1% isoflurane. One mouse was intravenously injected with MBBN (100 μL, 6 mg mL^-1^), while the other was not treated (contrast group). *In vivo* CT scanning was performed after injection of the MBBN at different time points (2h, 4 h, 24 h). All CT scans were performed using the PET/CT system (G8, Perkinelmer).

### *In vivo* MR imaging (MRI)

To evaluate the MRI contrast effect of MBBN, a phantom test was performed using a 3.0 T imaging system (GE, Discovery MR750 plus). The MBBN (200 μL, 2 mg mL^-1^) was intravenously injected into 4T1 tumor-bearing mice when the tumor volume reached around 140 mm^3^ after 4T1 cancer-cell implantation. The T_1_-weighted MR imaging of mice were acquired after the different intervals followed by MBBN administration.

### *In vivo* fluorescence imaging

To evaluate the biodistribution of MBBN in normal organs and tumor, mice bearing 4T1 cells were injected with 200 μL IR780-MBBN (2 mg mL^-1^, similar to PPy-MBBN synthesis method) through tail vein. Real-time NIR 780 nm fluorescent images of IR780-MBBN were recorded at different point by using IVIS Spectrum system. After the imaging completed, the mice were sacrificed, and the major organs with tumors were analysed for *ex vivo* fluorescence.

### Pharmacokinetic experiments

BGC-823 tumor-bearing nude mice were intravenously injected with MBBN solution (20 mg Kg^-1^, n=3). The 20 μL of blood was collected from sinus thrombosis at varied time points (0.5 h, 1 h, 2 h, 4 h, 8 h, 16 h, 24 h) after injection. The quantitative analysis of Mn and B element was measured by ICP-OES.

### *In vivo* tumor therapy

All healthy female Balb/c nude mice (4 weeks old) were purchased from Guangdong Medical Laboratory Animal Center and all the *in vivo* experiments followed the protocols approved by the Animal Care and Use Committee of the Shenzhen University. For BGC-823 tumor therapy, when the tumor size reached approximately 80-100 mm^3^ (designed as Day 0), the treatment was performed. Two experienced researchers randomly divided the mice into eight groups (n = 5 per group for BGC-823 tumor), which were *in situ* injected with 50 μL PBS without and with 808 nm laser irradiation (as two blank controls, Group 1 and Group 2), 5 mg Kg^-1^ MBN (Group 3), 5 mg Kg^-1^ MBBN (Group 4), 5 mg Kg^-1^ MBN with 808 nm laser irradiation with power density of 0.6 W cm^-2^ (Group 5), 5 mg Kg^-1^ MBBN with 808 nm laser irradiation with power density of 0.6 W cm^-2^ (Group 6), 5 mg Kg^-1^ MBN with 808 nm laser irradiation with power density of 1.0 W cm^-2^ (Group 7), 5 mg Kg^-1^ MBBN with 808 nm laser irradiation with power density of 1.0 W cm^-2^ (Group 8), on Day 1, 2. Group 2, 5, 6, 7 and 8 were treated with 808 nm laser for 5 min at tumor sites after 1 h injection. The body weight and tumor volume (V = (ab^2^)/2, where a and b refer to the largest length and width of tumor, respectively) of each mouse were recorded every other day. The mice were humanely killed after 21 days of treatment and all the tumors were collected.

### Histological stained analysis

All mice were humanely killed and main organs (heart, liver, spleeny, lung and kidney) and tumors were harvested after 21 days treatment, fixed in a 4% polyoxymethylene solution, and embedded in paraffin for H&E, TUNEL and Ki-67 staining.

### *In vivo* Biocompatibility Assay

Healthy female Balb/c mice (n = 4 in each group) were adopted for evaluation. The MBN and MBBN (10 mg kg^-1^) were intravenously injected into the mice. The mice without any treatment were used as the control group. After 1 week of feeding, the mice were sacrificed and their blood samples were taken out for the various blood indexes assessment.

## Supplementary Material

Supplementary figures and tables. The Supporting Information is available free of charge on the website at DOI: xxxx.

## References

[1] Tang Q, Zhou Z (2013). Graphene-analogous low-dimensional materials. Prog Mater Sci.

[2] Yang B, Chen Y, Shi J (2018). Material chemistry of two-dimensional inorganic nanosheets in cancer theranostics. Chem.

[3] Tan C, Cao X, Wu X, He Q, Yang J, Zhang X (2017). Recent advances in ultrathin two-dimensional nanomaterials. Chem Rev.

[4] Mannix AJ, Kiraly B, Hersam MC, Guisinger NP (2017). Guisinger, Synthesis and chemistry of elemental 2D materials. Nat Rev Chem.

[5] Cai G, Yu Z, Ren R, Tang D (2018). Exciton-plasmon interaction between AuNPs/graphene nanohybrids and CdS quantum dots/TiO_2_ for photoelectrochemical aptasensing of prostate-specific antigen. ACS Sens.

[6] Novoselov KS, Geim AK, Morozov SV, Jiang D, Zhang Y, Dubonos SV (2004). Electric field effect in atomically thin carbon films. Science.

[7] Manzeli S, Ovchinnikov D, Pasquier D, Yazyev OV, Kis A (2017). 2D transition metal dichalcogenides. Nat Rev Mater.

[8] Takada K, Sakurai H, Takayama-Muromachi E, Izumi F, Dilanian RA, Sasaki T (2003). Superconductivity in two-dimensional CoO_2_ layers. Nature.

[9] Cai G, Yu Z, Tong P, Tang D (2019). Ti_3_C_2_ MXene quantum dot-encapsulated liposomes for photothermal immunoassays using a portable near-infrared imaging camera on a smartphone. Nanoscale.

[10] Liu Z, Lin H, Zhao M, Dai C, Zhang S, Peng W (2018). 2D superparamagnetic tantalum carbide composite MXenes for efficient breast-cancer theranostics. Theranostics.

[11] Lv S, Tang Y, Zhang K, Tang D (2018). Wet NH_3_-triggered NH_2_-MIL-125(Ti) structural switch for visible fluorescence immunoassay impregnated on paper. Anal Chem.

[12] Zhan G, Fan L, Zhao F, Huang Z, Chen B, Yang X (2019). Fabrication of ultrathin 2D Cu-BDC nanosheets and the derived integrated MOF nanocomposites. Adv Funct Mater.

[13] Li B, Wang X, Chen L, Zhou Y, Dang W, Chang J (2018). et al. Ultrathin Cu-TCPP MOF nanosheets: A new theragnostic nanoplatform with magnetic resonance/near-infrared thermal imaging for synergistic phototherapy of cancers. Theranostics.

[14] Qiu Z, Shu J, Tang D (2018). Plasmonic resonance enhanced photoelectrochemical aptasensors based on g-C_3_N_4_/Bi_2_MoO_6_. Chem Commun.

[15] Liu Z, Ma L, Shi G, Zhou W, Gong Y, Lei S (2013). In-plane heterostructures of graphene and hexagonal boron nitride with controlled domain sizes. Nat Nanotechnol.

[16] Tao W, Zhu X, Yu X, Zeng X, Xiao Q, Zhang X (2017). Black phosphorus nanosheets as a robust delivery platform for cancer theranostics. Adv Mater.

[17] Boustani I in Chemical Modelling: Applications and Theory, The Royal Society of Chemistry. 2011; 8: 1-44.

[18] Kaner RB, Gilman JJ, Tolbert SH (2005). Designing superhard materials. Science.

[19] Kim H, Wang Z, Alshareef HN (2019). MXetronics: Electronic and photonic applications of MXenes. Nano Energy.

[20] Chaudhari NK, Jin H, Kim B, Baek DS, Joo SH, Lee K (2017). MXene: an emerging two-dimensional material for future energy conversion and storage applications. J Mater Chem A.

[21] Han X, Jing X, Yang D, Lin H, Wang Z, Ran H (2018). Therapeutic mesopore construction on 2D Nb_2_C MXenes for targeted and enhanced chemo-photothermal cancer therapy in NIR-II biowindow. Theranostics.

[22] Medvedeva NI, Ivanovskii AL, Medvedeva JE, Freeman A (2001). Electronic structure of MgB_2_ and related binary and ternary borides. Phys Rev B.

[23] Jiang Z, Wang P, Jiang X, Zhao JJ (2018). MBene (MnB): A new type of 2D metallic ferromagnet with high Curie temperature. Nanoscale Horiz.

[24] Alameda LT, Moradifar P, Metzger ZP, Alem N, Schaak RE (2018). Topochemical deintercalation of Al from MoAlB: Stepwise etching pathway, layered intergrowth structures, and two-dimensional MBene. J Am Chem Soc.

[25] Xu BZ, Beckman SP (2016). Quantum confinement induced band gaps in MgB_2_ nanosheets.

[26] Lundquist N, Myers HP, Westin R (1962). The paramagnetic properties of the monoborides of V, Cr, Mn, Fe, Co and Ni. Philos Mag.

[27] Fan M, Wen Y, Ye D, Jin Z, Zhao P, Chen D (1900). Acid-responsive H_2_-releasing 2D MgB_2_ nanosheet for therapeutic synergy and side effect attenuation of gastric cancer chemotherapy. Adv Healthc Mater.

[28] Naguib M, Mochalin VN, Barsoum MW, Gogotsi Y (2014). MXenes: A new family of two-dimensional materials. Adv Mater.

[29] Barsoum MW (2000). The M_N+1_AX_N_ phases: A new class of solids: Thermodynamically stable nanolaminates. Prog Solid State Chem.

[30] Naguib M, Kurtoglu M, Presser V, Lu J, Niu J, Heon M (2011). Two-dimensional nanocrystals produced by exfoliation of Ti_3_AlC_2_. Adv Mater.

[31] Jian T, Cheung LF, Chen TT, Wang LS (2017). Bismuth-boron multiple bonding in BiB_2_O^-^ and Bi_2_B^-^. Angew Chem Int Ed.

[32] Romanescu C, Galeev TR, Li WL, Boldyrev AI, Wang LS (2013). Transition-metal-centered monocyclic boron wheel clusters (M@Bn): A new class of aromatic borometallic compounds. Acc Chem Res.

[33] Yang K, Zhang S, Zhang G, Sun X, Lee ST, Liu Z (2010). Graphene in mice: ultrahigh *in vivo* tumor uptake and efficient photothermal therapy. Nano Lett.

[34] Lin H, Wang Y, Gao S, Chen Y, Shi J (2018). Theranostic 2D tantalum carbide (MXene). Adv Mater.

[35] Deng L, Cai X, Sheng D, Yang Y, Strohm EM, Wang Z (2017). A laser-activated biocompatible theranostic nanoagent for targeted multimodal imaging and photothermal therapy. Theranostics.

[36] Roper DK, Ahn W, Hoepfner M (2007). Microscale heat transfer transduced by surface plasmon resonant gold nanoparticles. J Phys Chem C.

[37] Zhang L, Sheng D, Wang D, Yao Y, Yang K, Wang Z (2018). Bioinspired multifunctional melanin-based nanoliposome for photoacoustic/magnetic resonance imaging-guided efficient photothermal ablation of cancer. Theranostics.

[38] Lin H, Wang X, Yu L, Chen Y, Shi J (2017). Two-dimensional ultrathin MXene ceramic nanosheets for photothermal conversion. Nano Lett.

[39] Lin H, Gao S, Dai C, Chen Y, Shi J (2017). A Two-Dimensional Biodegradable Niobium Carbide (MXene) for Photothermal Tumor Eradication in NIR-I and NIR-II Biowindows. J Am Chem Soc.

[40] Luo Y, Li Z, Zhu C, Cai X, Qu L, Du D (2018). Graphene-like metal-free 2D nanosheets for cancer imaging and theranostics. Trends Biotechnol.

[41] Peng L, Mei X, He J, Xu J, Zhang W, Liang R (2018). Monolayer nanosheets with an extremely high drug loading toward controlled delivery and cancer theranostics. Adv Mater.

[42] Eliaz RE, Szoka FC (2001). Liposome-encapsulated doxorubicin targeted to CD44. Cancer Res.

[43] Pure E, Cuff CA (2001). A crucial role for CD44 in inflammation. Trends Mol Med.

[44] Zheng JZ, Liu JB, Dunne M, Jaffray DA, Allen C (2007). *In vivo* performance of a liposomal vascular contrast agent for CT and MR-based image guidance applications. Pharm Res.

[45] Smith BR, Gambhir SS (2017). Nanomaterials for *in vivo* imaging. Chem Rev.

[46] Li C, Yang X, Zhang M, Song Y, Cheng K, An J *In vivo* imaging-guided nanoplatform for tumor targeting delivery and combined chemo-, gene- and photothermal therapy. 2018; 8: 5662-75.

[47] Gao K, Tu W, Yu X, Ahmad F, Zhang X, Wu W (2019). W-doped TiO_2_ nanoparticles with strong absorption in the NIR-II window for photoacoustic/CT dual-modal imaging and synergistic thermoradiotherapy of tumors. Theranostics.

[48] Rabin O, Perez JM, Grimm J, Wojtkiewicz G, Weissleder R (2006). An X-ray computed tomography imaging agent based on long-circulating bismuth sulphide nanoparticles. Nat Mater.

[49] Yang L, Wang J, Yang S, Lu Q, Li P, Li N (2019). Rod-shape MSN@MoS_2_ nanoplatform for FL/MSOT/CT imaging-guided photothermal and photodynamic therapy. Theranostics.

