# Supplementary material for: Coordination-induced exfoliation to monolayer Bi-anchored MnB2 nanosheets for multimodal imaging-guided photothermal therapy of cancer
Source: Theranostics. 2020 Jan 1;10(4):1861–72. doi: 10.7150/thno.39715 (PMC6993225; doi:10.7150/thno.39715)
Supplement: Supplementary file 1 — Supplementary figures and tables. The Supporting Information is available free of charge on the website at DOI: xxxx. [file thnov10p1861s1.pdf]

# Supporting Information:

# Coordination-induced exfoliation to monolayer Bi-anchored $MnB_2$ nanosheets for multimodal imaging-guided photothermal therapy of cancer

Zhaokui Jin,† Danyang Chen,† Penghe Zhao, Yanyuan Wen, Mingjian Fan, Gaoxin Zhou, Yingshuai Wang, Qianjun He*

Guangdong Provincial Key Laboratory of Biomedical Measurements and Ultrasound Imaging, National-Regional Key Technology Engineering Laboratory for Medical Ultrasound, School of Biomedical Engineering, Health Science Center, Shenzhen University

No. 1066 Xueyuan Road, Nanshan District, Shenzhen 518060, Guangdong, China

* Correspondence to: Qianjun He (nanoflower@126.com)

† These coauthors contributed equally to this work: Zhaokui Jin and Danyang Chen.

## Experimental Procedures

**Materials:** Polyvinylpyrrolidone (PVP, Mw=55000), anhydrous acetic acid, hyaluronic acid (HA) were purchased from Sigma-Aldrich. Hydrogen peroxide ($H_2O_2$, 30%), ethyl alcohol, ethylene glycol (EG) were purchased from Aladdin Industrial Corporation. Manganese boride was purchased from JinZhou Haixin Metal Materials Co. Ltd, (Liaoning, China). 4',6-diamidino-2-phenylindole (DAPI), Propidium Iodide, Calcein-AM were purchased from GL biochem Ltd (Shanghai). The CCK-8 kit was purchased from Beyotime Biotechnology Co. Ltd,.

**Characterization:** The morphology and structure of BPN-based complexes were measured by scanning electron microscope (SEM), transmission electron microscopy (TEM) and atomic force microscope (AFM). SEM images were obtained on an APREO-S (FEI). TEM images were obtained on a HT-7700 (HITACHI). The mapping of Mn/B/Bi elements was collected for co-localization of various elements in the MBBN on a JEOL-F200. AFM images were obtained on a BRUKER-MultiMode 8. The phase structures of MnB-based complexes were characterized by powder X-ray diffraction (PXRD) using a M21X diffractometer (Cu Kα, λ = 1.54056 Å) operated at 40 kV and 200 mA. The diffraction patterns were collected with a scanning range of 5°−80° at room temperature. The composition of MnB-based complexes was tested by attenuated total reflectance Fourier transform infrared spectroscopy (ATR-FTIR). Concentrated sample solutions were dropped on the universal diamond ATR sampling accessory and slowly dried by blowing with nitrogen gas. FT-IR spectra were then collected on a Thermo-Nicolet Nexus 670 ATR-IR spectrometer. The UV adsorption spectra of sample solutions (200–1000 nm) were recorded at room temperature on a Genesys 10S UV-Vis spectrophotometer (Thermo Sci.). XPS spectra of MnB-based complexes were used to confirm the coordination structure, which were performed on an ultra-high vacuum system with Mg Ka (1253.6 eV) as excitation source. The CLSM image were obtained using a LSM880 camera, ZEISS, German.

The photothermal conversion efficiencies (η) of MBN and MBBN were calculated as followed:
Following Roper's report,[1] the photothermal conversion efficiency (η) of $MBBN_{0.1}$ was calculated using the following equation

$$\eta = \frac{hS\left(T_{\max} - T_{surr}\right) - Q_{Dis}}{I\left(1 - 10^{-A_{808}}\right)} \quad (1)$$

Where *h* (mW/(m$^2$·°C)) is heat transfer coefficient, *S* (m$^2$) is the surface area of the container, and $T_{max}$ is the equilibrium temperature, $T_{surr}$ is the ambient temperature. In this experiment, $T_{max}-T_{surr}$ was 38.8 °C according to Figure S7. The $Q_{Dis}$ (mW) expresses the heat from light absorbed by the cuvette sample walls itself and it was measure to be 4.93 mW independently using a quartz cuvette cell containing aqueous samples without $MBBN_{0.1}$ ( $m_{pp}$=0.519 g, $C_{pp}$=1.926). *I* is the incident laser power (1 W) and $A_{808}$ is the absorbance (0.33) at 808 nm.
To get the *hS*, *θ* is introduced using the maximum system temperature, $T_{max}$

$$\theta = \frac{T - T_{surr}}{T_{\max} - T_{surr}} \quad (2)$$

and a sample system time constant $\tau_s$

$$\tau_s = \frac{\sum_i m_i C_{p,i}}{hS} \qquad (3)$$

according to the following expression[1-2, 7]

$$t = -\tau_s \ln(\theta) \qquad (4)$$

$\tau_s$ was determined to be 288.07 s thus *hS* was deduced to be 8.75 mW/°C (substituted $m$ = 0.6 g, $C$ = 4.2 J/g·K in Equation (3)).

Finally, the photothermal conversion efficiency (η) of $MBBN_{0.1}$ was calculated to be 59.4% from Equation (1). In the same way, that of MBN was 42.4%.

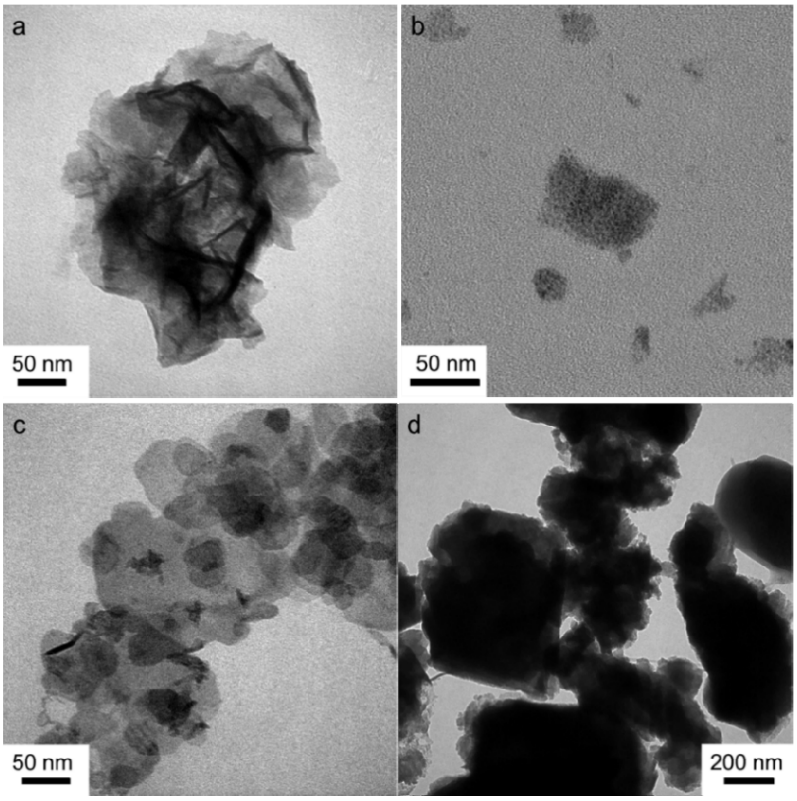


**Figure S1.** Typical TEM images of MBN (a), $MBBN_{0.1}$ (b), $MBBN_{0.2}$ (c), and $MBBN_{0.4}$ (d).

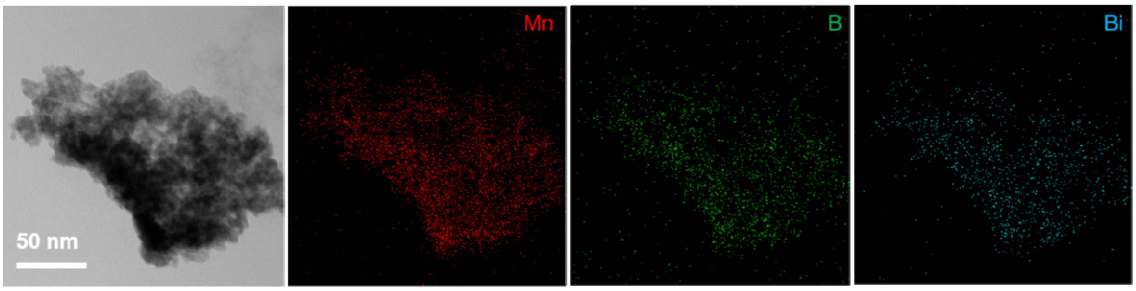


**Figure S2.** TEM image and corresponding EDS element mapping images of MBBN.

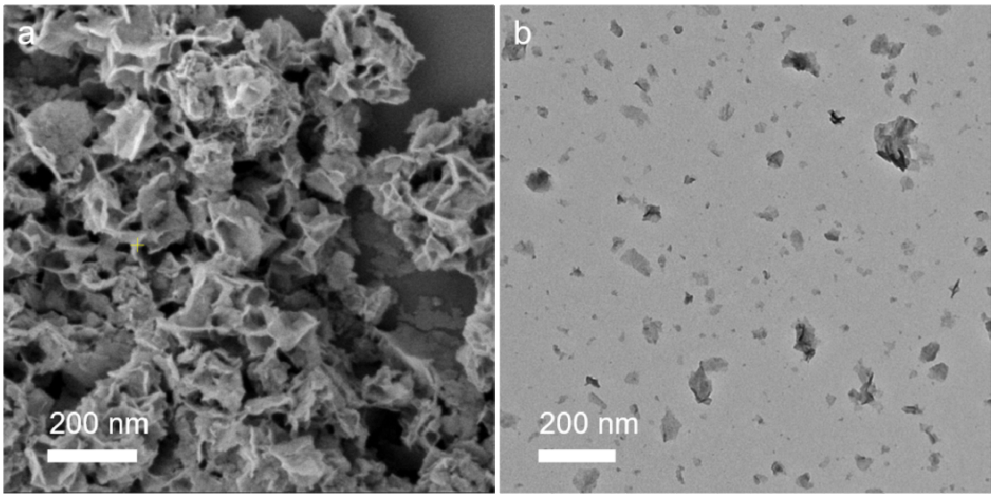


**Figure S3.** Typical SEM (a) and TEM (b) images of MBBN.

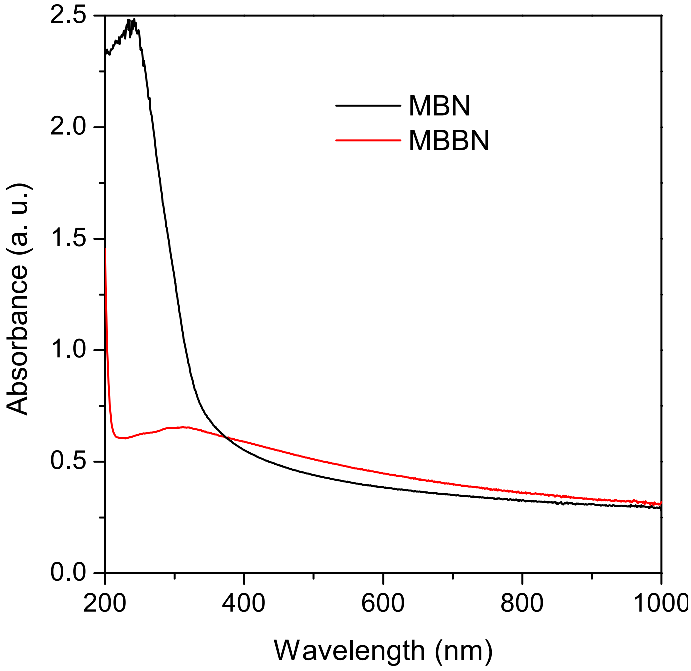


**Figure S4.** UV-VIS-NIR spectra of MBN and MBBN at the concentration of 100 μg $mL^{-1}$.

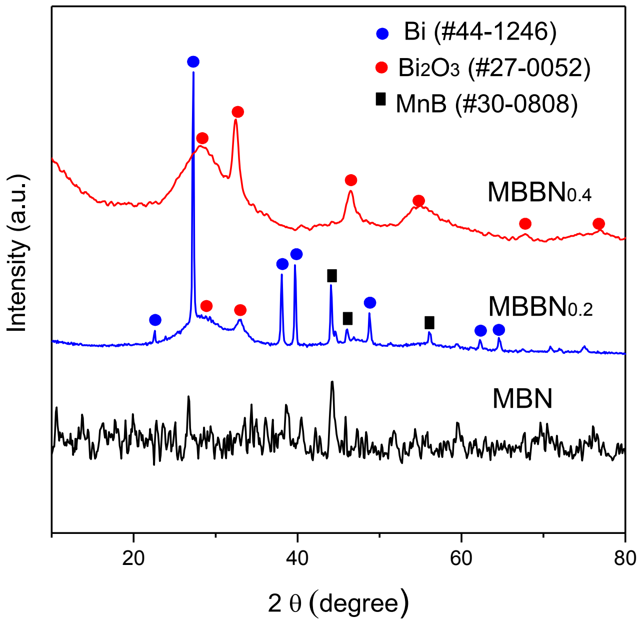


**Figure S5.** XRD patterns of MBN, $MBBN_{0.2}$ and $MBBN_{0.4}$.

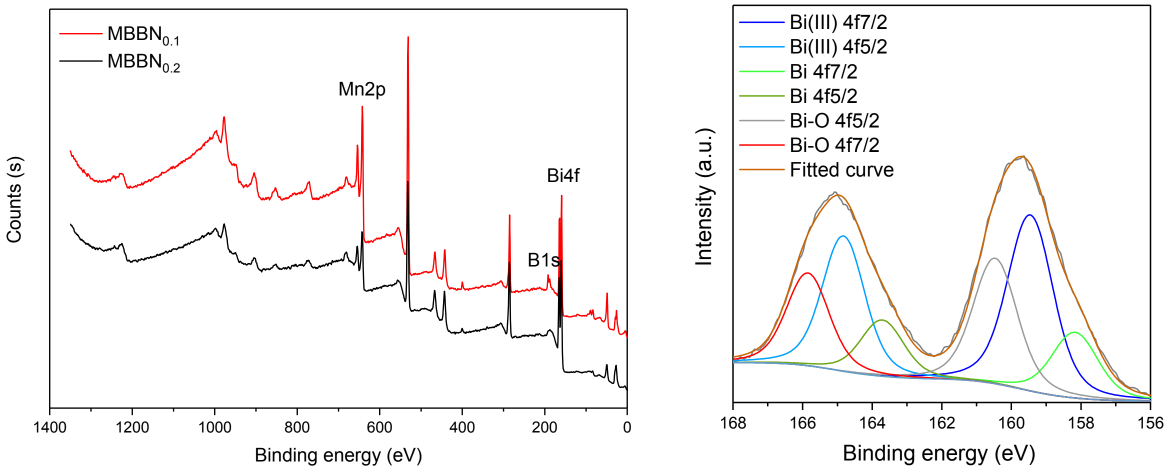


**Figure S6.** XPS spectra of $MBBN_{0.1}$ and $MBBN_{0.2}$ (left), and high resolution Bi 4f spectra of $MBBN_{0.2}$ (right).

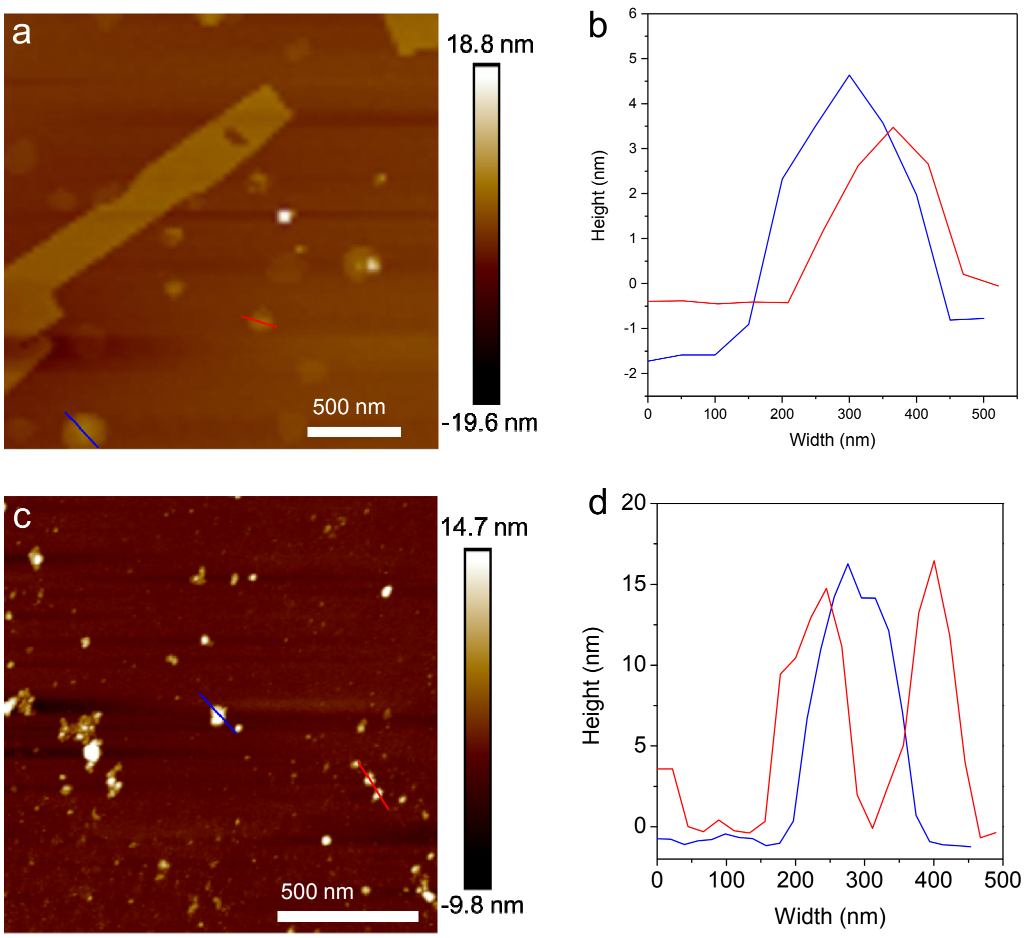


**Figure S7.** Typical AFM images of MBN (a) and $MBBN_{0.2}$ (c), and the corresponding height distribution curves (b, d).

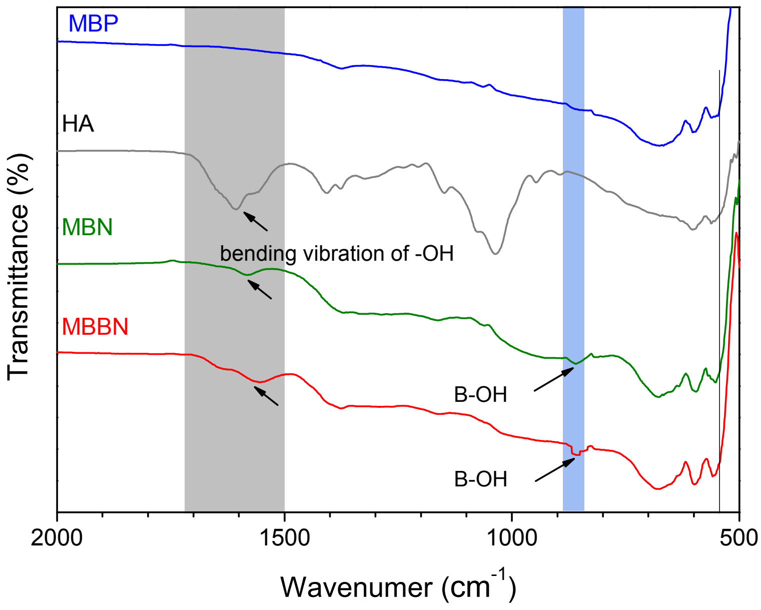


**Figure S8.** ATR-FTIR spectra of MBP, HA, MBN and MBBN.

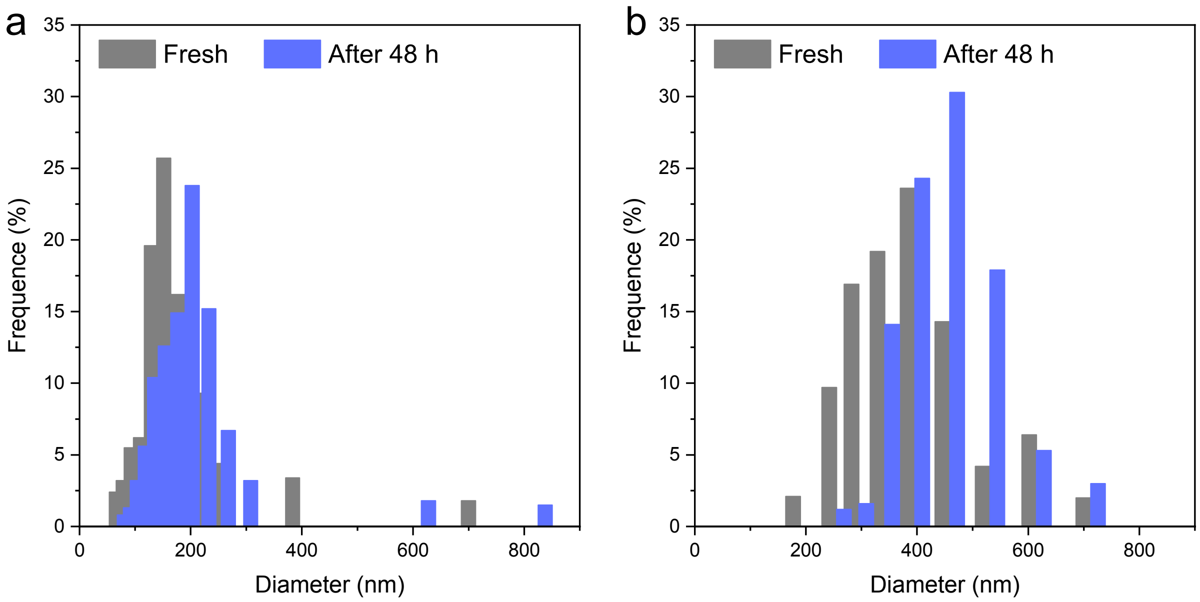


**Figure S9.** DLS data of MBBN (a) and MBN (b) at different time points.

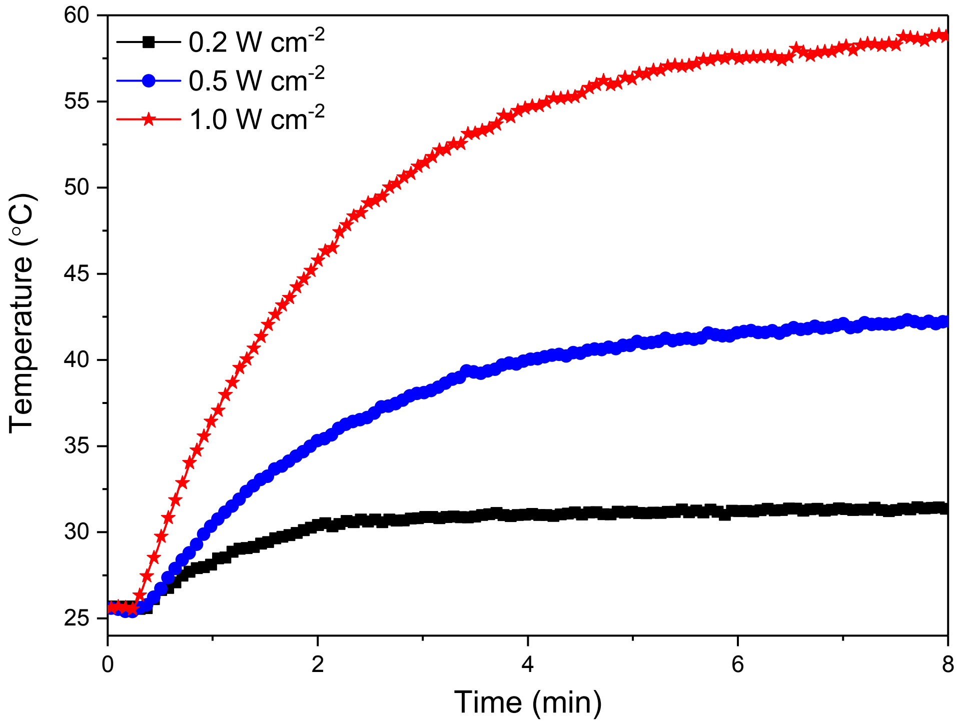


**Figure S10.** Photothermal heating curves of MBN (100 μg $mL^{-1}$) under the irradiation with 808 nm laser at varied power density (0.2, 0.5, 1.0 W $cm^{-2}$).

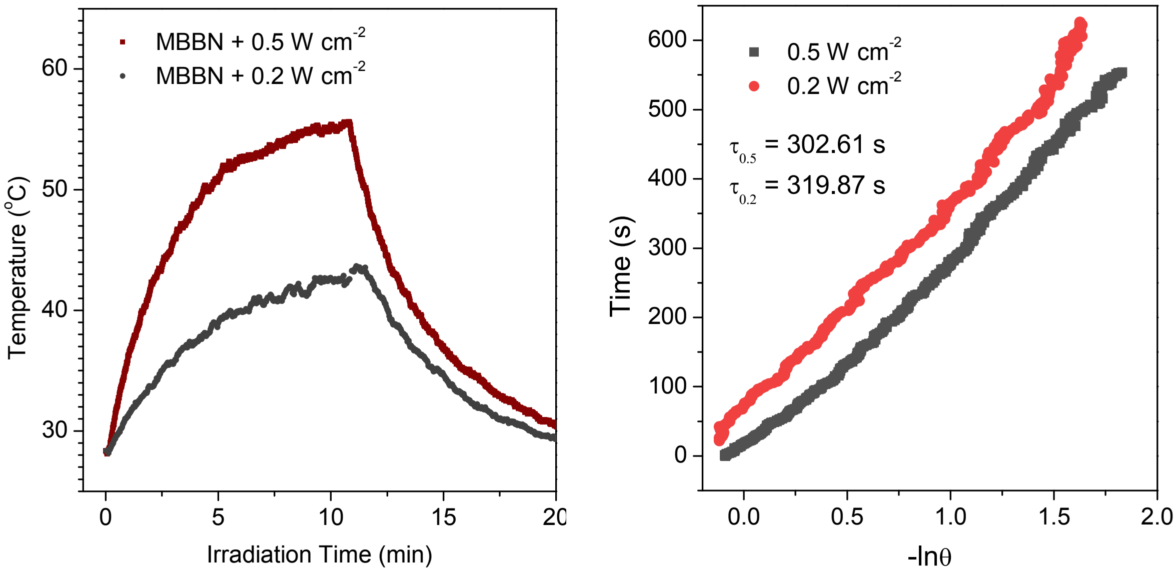


**Figure S11.** Photothermal effect of MBN aqueous dispersion (200 μg/mL) under the irradiation of 808 nm laser at different power densities, and the laser was turned off after irradiation for 10 min (left). The time constant for heat transfer from the system was determined by applying the linear time data from the cooling period (after 10 min) versus negative natural logarithm of the driving force temperature obtained from the cooling stage (right). The results from Figure 2b and Figure S11 show that the photothermal conversion efficiencies under 1.0, 0.5 and 0.2 W/cm$^2$ were calculated to be 59.4%, 57.7% and 61.4%, respectively. The slight difference among them possibly results from the influence of faint surrounding temperature change.

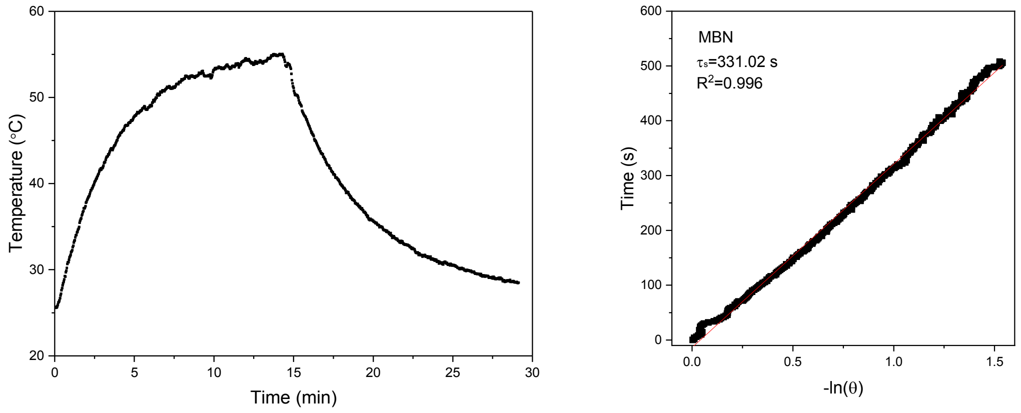


**Figure S12.** Photothermal effect of MBN aqueous dispersion (100 μg/mL) under irradiation of 808 nm laser with the power density of 1 W cm$^{-2}$ and the laser was turned off after irradiation for 14 min (left). The time constant for heat transfer from the system was determined to be $\tau_s$ = 331.02 s by applying the linear time data from the cooling period (after 14 min) versus negative natural logarithm of the driving force temperature obtained from the cooling stage (right).

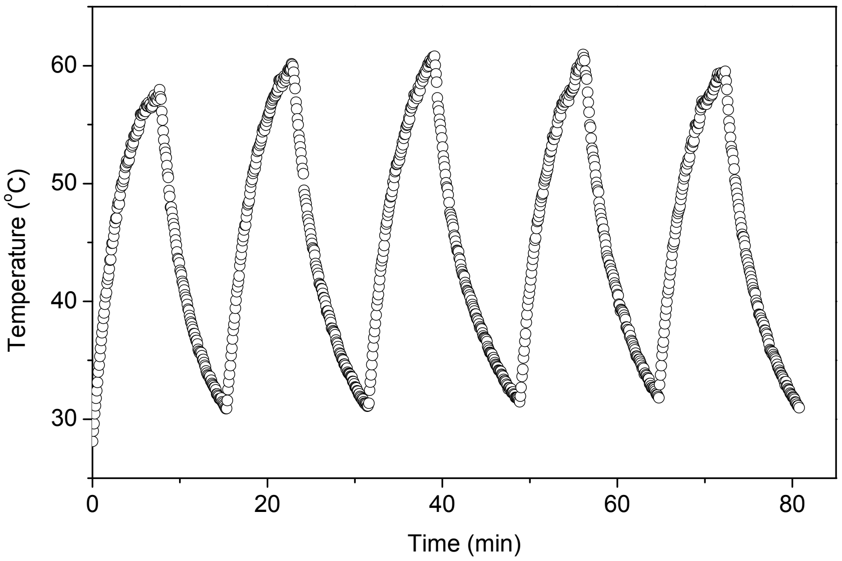


**Figure S13.** Photothermal conversion stability of MBN for five laser on/off cycles (200 μg/mL /mL, 1.0 W $cm^{-2}$).

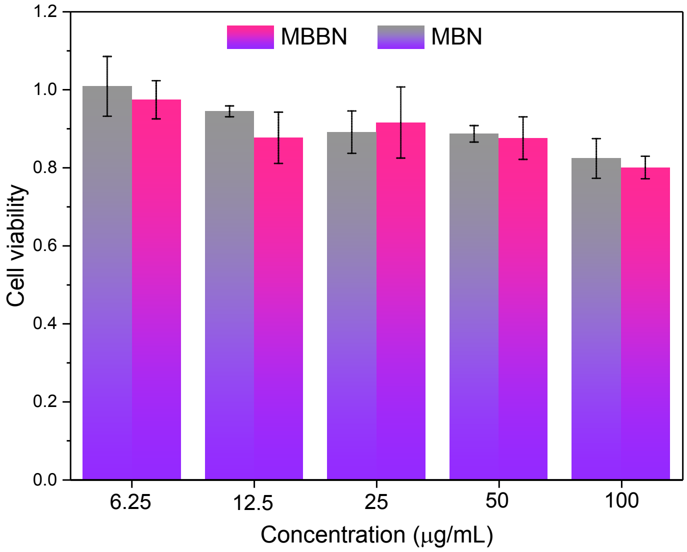


**Figure S14.** Relative viabilities of BGC-823 cells after incubation with MBN and MBBN of varied concentrations (6.25, 12.5, 25, 50, and 100 μg $mL^{-1}$) for 48 h.

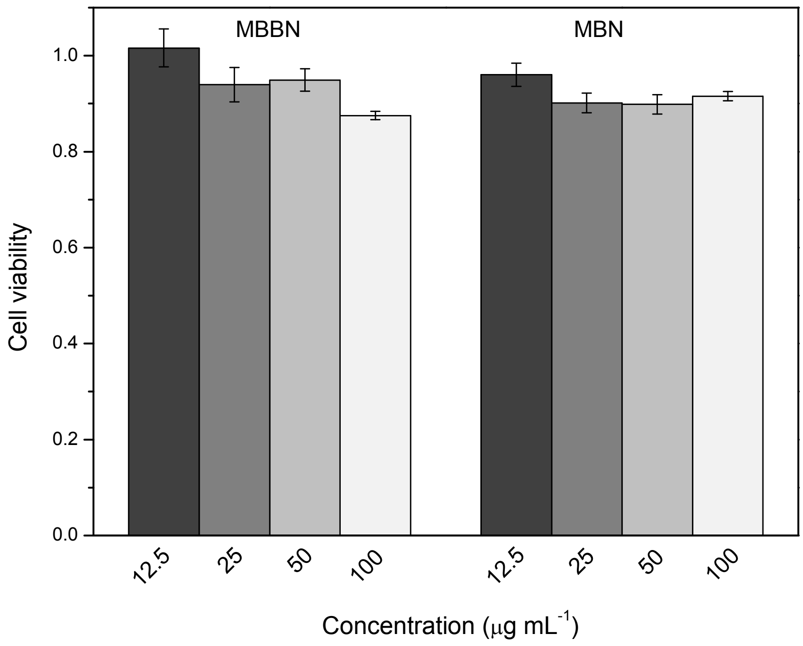


**Figure S15.** Relative viabilities of Hek-293t cells after incubation with MBN and MBBN of varied concentrations (12.5, 25, 50, and 100 μg mL$^{-1}$) for 24 h.

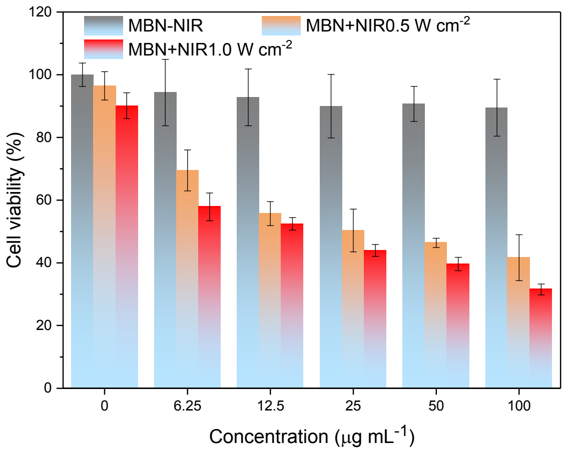


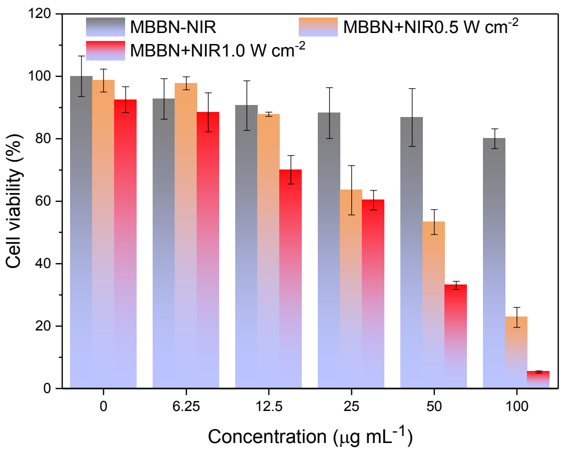


**Figure S16.** Relative viabilities of BGC-823 cells after treatment with varied concentrations of MBN (left) or MBBN (right) at different laser power densities (0, 0.5, 1.0 W cm$^{-2}$).

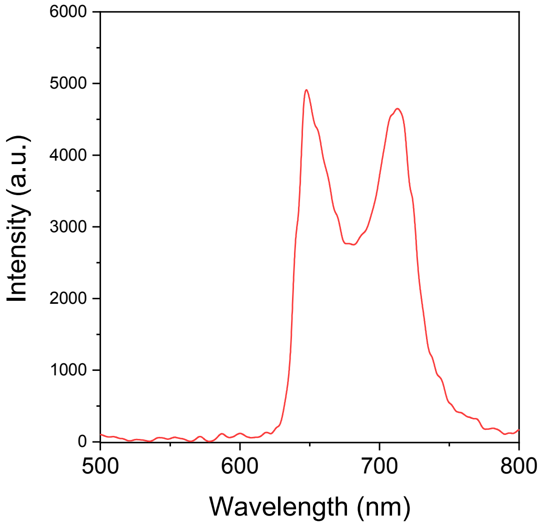


**Figure S17.** Fluorescent spectrum of PPy-MBBN (excitation wavelength at 425 nm).

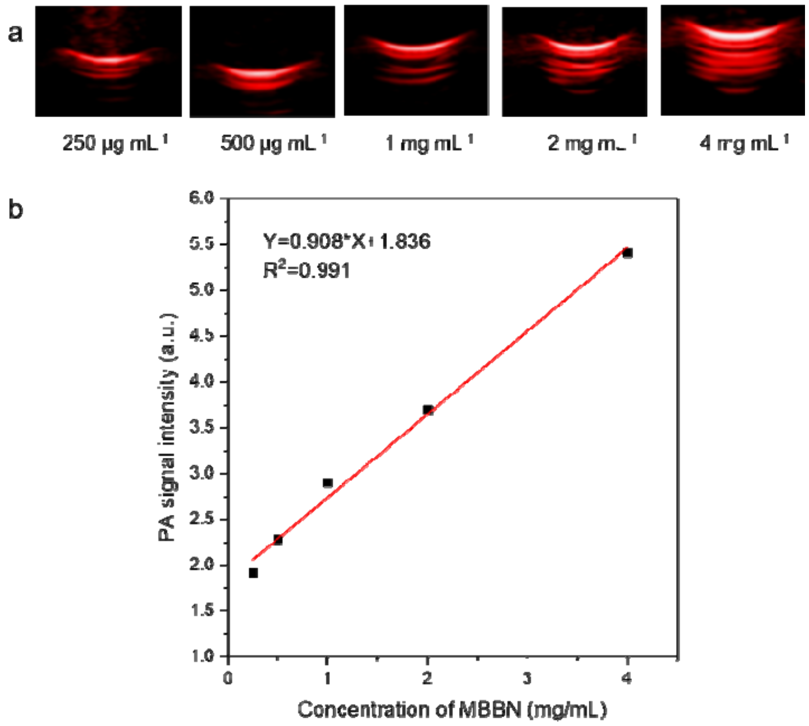


**Figure S18.** The dependence of in vitro PA imaging signal of MBBN solution on its concentration, and (b) the corresponding quantitative analysis.

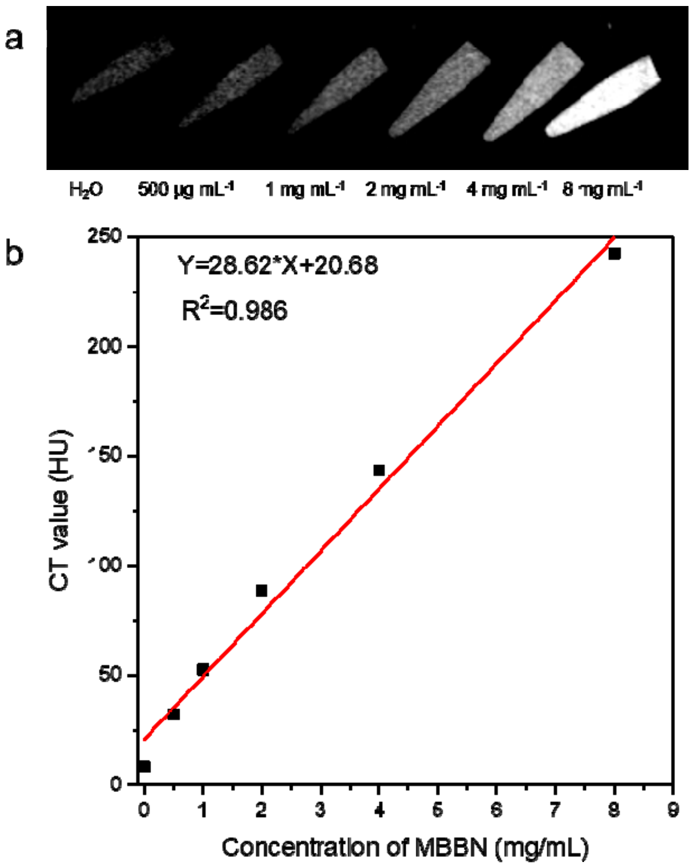


**Figure S19.** (a) In vitro CT images of MBBN with different concentrations. (b) The corresponding quantitative analysis.

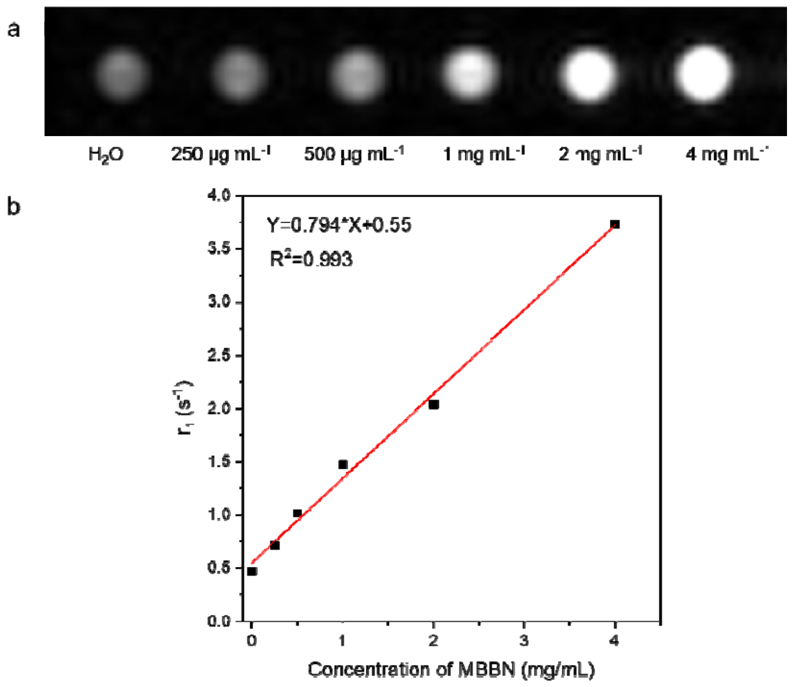


**Figure S20.** (a) In vitro $T_1$-weighted MR images obtained from MBBN with various concentrations. (b) the corresponding plot of $r_1$ relaxation rate.

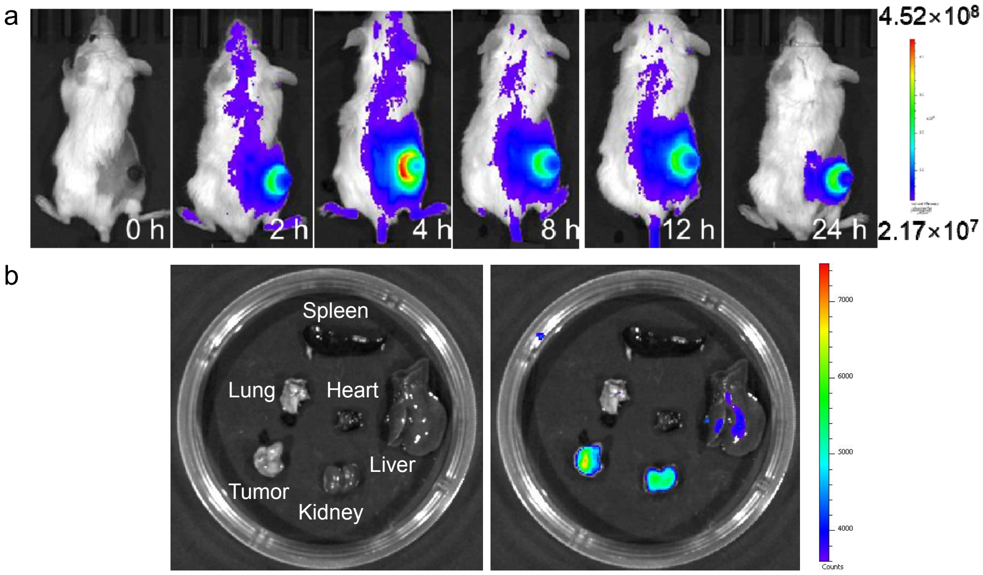


**Figure S21.** (a) *In vivo* fluorescence images of 4T1 tumor-bearing mouse obtained at different time points post-injection of MBBN-IR780. (b) *Ex vivo* fluorescence images of main organs and tumor after injection for 24 h.

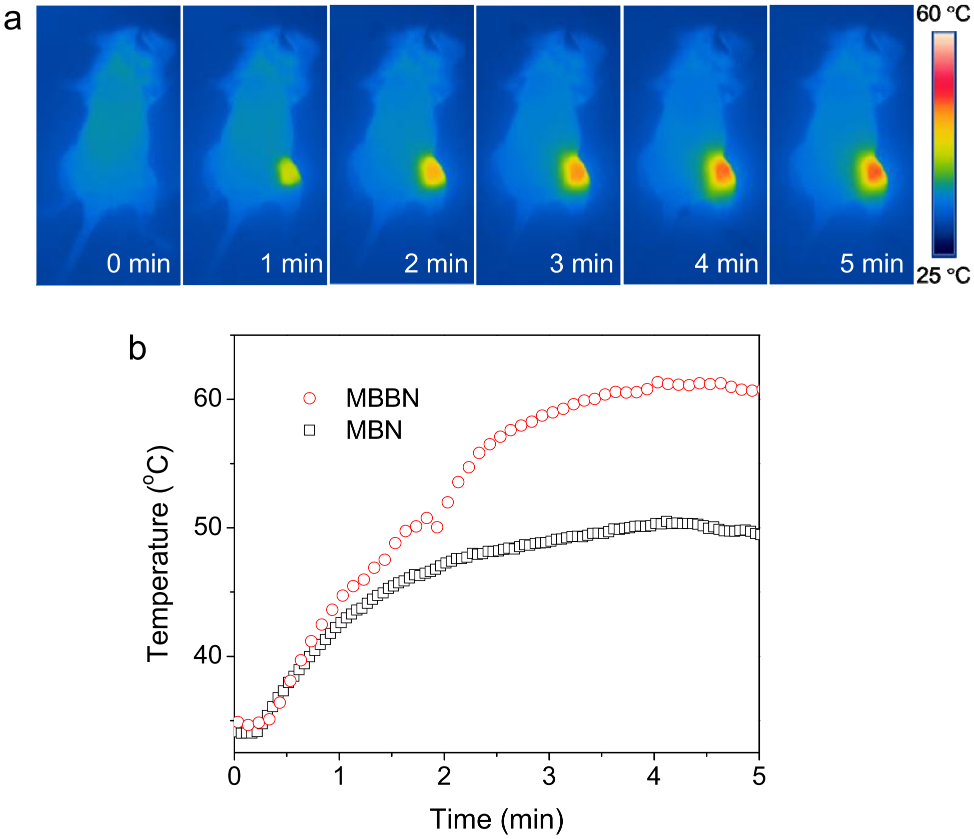


**Figure S22.** (a) Infrared thermographic images of 4T1-tumor-bearing mice in groups of MBN (20 mg $kg^{-1}$) at different time points during laser irradiation. (b) The corresponding time-dependent temperature changes at the tumor sites of 4T1-tumor-bearing mice during laser irradiation.

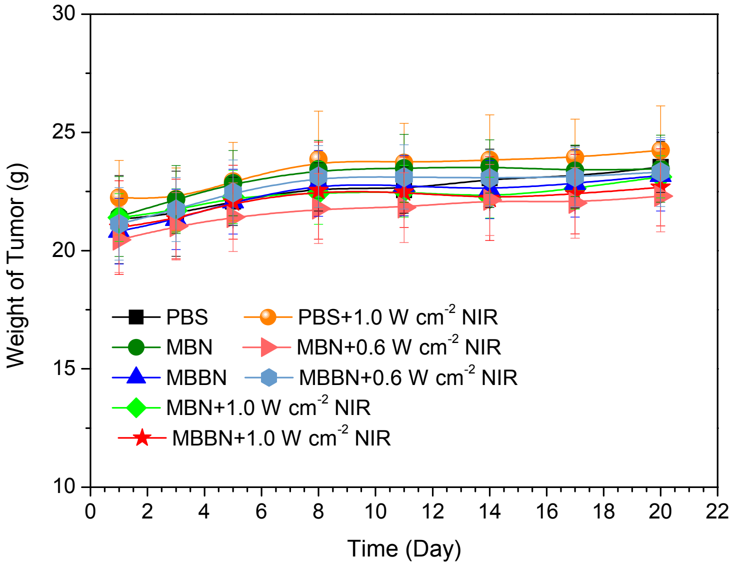


**Figure S23.** Body-weight changes in different groups during 21 days treatment.

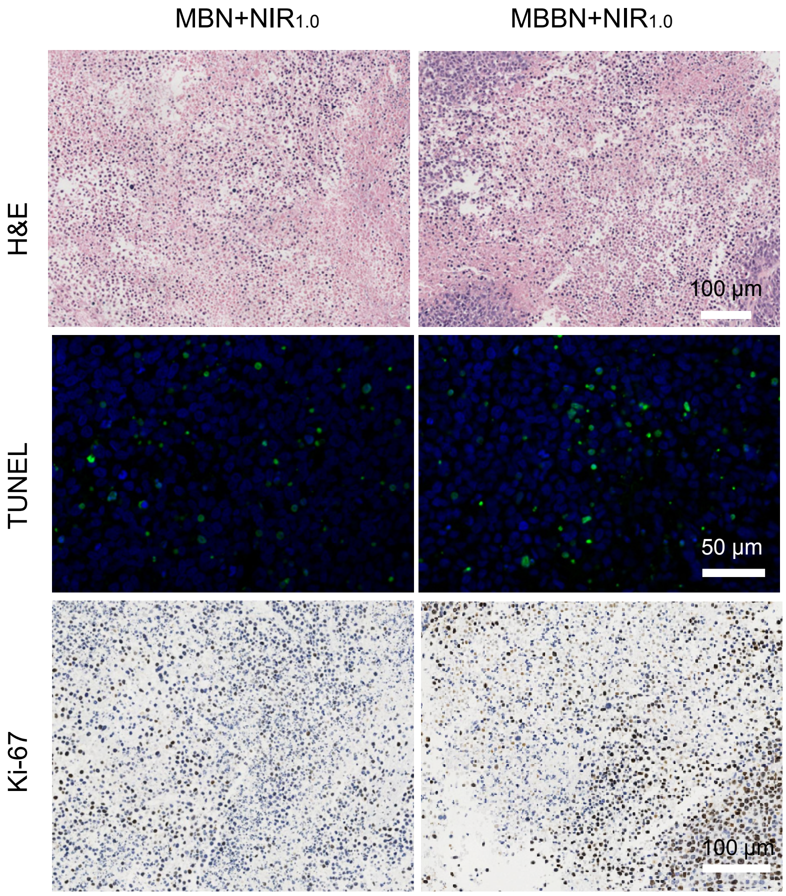


**Figure S24.** Histological analysis including H&E staining for pathological changes in tumor tissues to reveal the effectiveness of *in vivo* photothermal therapy, the TUNEL staining for cell apoptosis in tumor sections, and Antigen Ki-67 immunofluorescence staining for cellular proliferation in tumor sections.

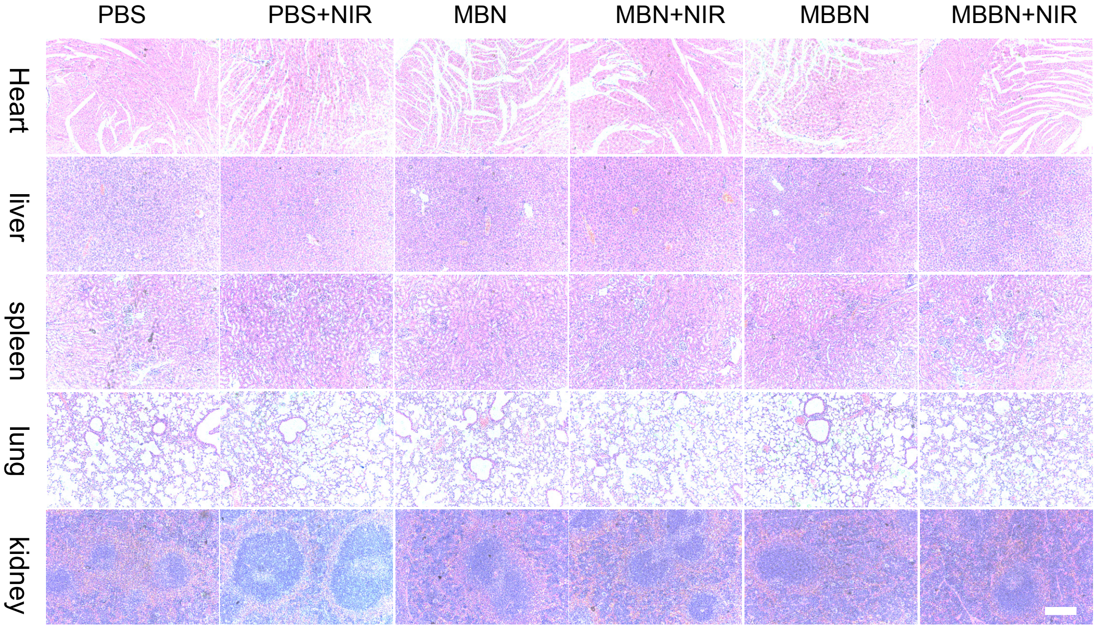


**Figure S25.** H&E staining of major organs from different groups after 21 days treatment (Scale bar, 200 μm).

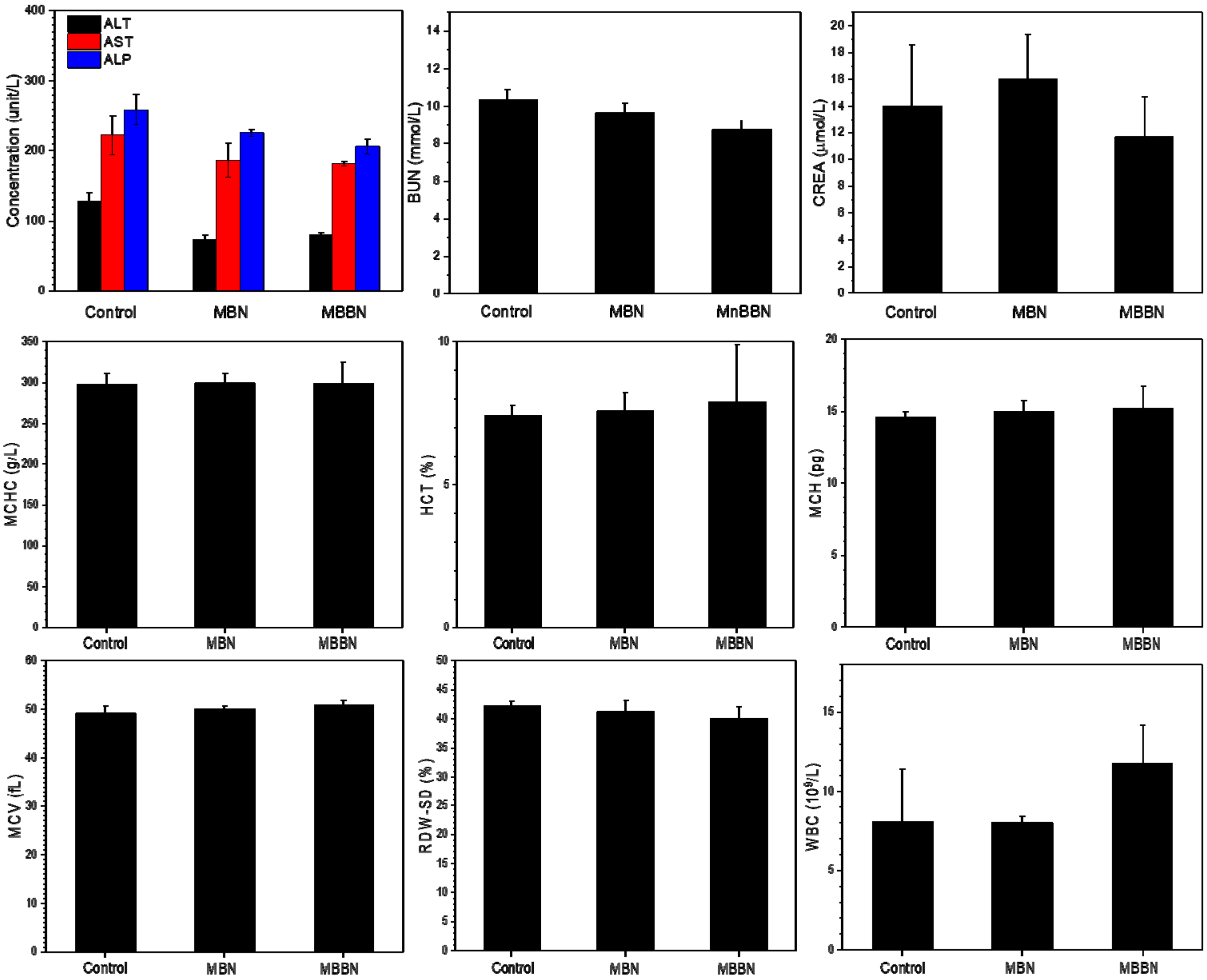


**Figure S26.** Serum biochemistry analysis of mice after various treatments. The results show mean and

standard deviation of kidney functions and liver functions markers (ALT, AST and ALP levels, BUN level, CREA levels of mice in all groups, reflecting the kidney functions and liver functions.), Hemoglobin, HCT, MCH, MCV, RDW-SD, WBC levels of mice in all groups. Mean values and error bars are defined as mean and s.d., respectively.

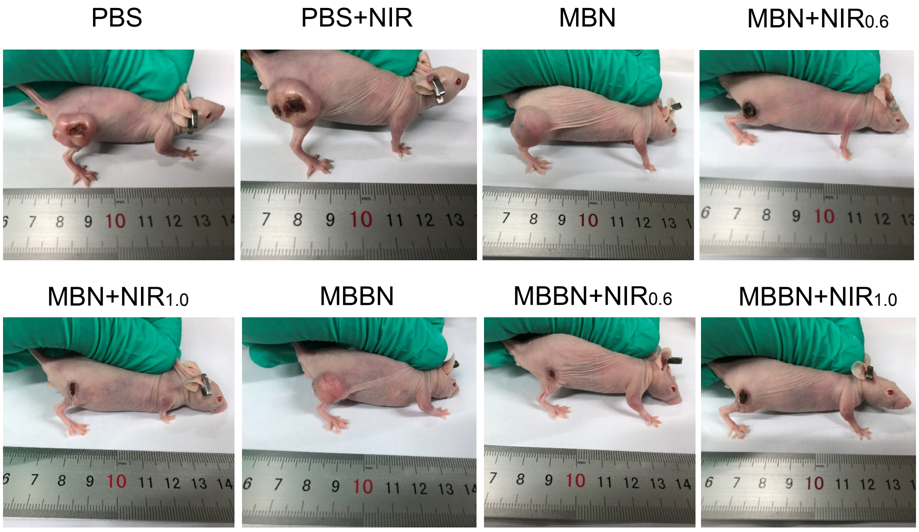


**Figure S27.** Photographs of BGC-823 tumor-bearing nude mice and its tumor regions in 21 d after different treatments.

**Table S1.** Elemental content of the product measured by ICP.

| | Mn | Bi |
|---|---|---|
| MBN | 13.5 μM | / |
| $MBBN_{0.1}$ | 9.94 μM | 0.52 μM |
| $MBBN_{0.2}$ | 5.71 μM | 1.08 μM |
| $MBBN_{0.4}$ | 2.25 μM | 2.79 μM |

**References**

1. Roper DK, Ahn W, Hoepfner M. Microscale heat transfer transduced by surface plasmon resonant gold nanoparticles, J. Phys. Chem. C. 2007; 111: 3636-3641.
